# Exploring Greek actinobacterial biodiversity for the discovery of bioactive metabolites with skin antiaging potential

**DOI:** 10.3389/fmicb.2025.1649460

**Published:** 2025-10-09

**Authors:** Konstantinos Gaitanis, Eirini Gkogkou, Paris Laskaris, Nikolaοs Tsafantakis, Despoina D. Gianniou, Stavroula I. Kaili, Georgia C. Ntroumpogianni, Aikaterini Theodosopoulou, Nikola Milic, Dimitris G. Hatzinikolaou, Nikolas Fokialakis, Ioannis P. Trougakos, Amalia D. Karagouni

**Affiliations:** ^1^Division of Pharmacognosy and Chemistry of Natural Products, Department of Pharmacy, National and Kapodistrian University of Athens, Athens, Greece; ^2^Division of Cell Biology and Biophysics, Department of Biology, National and Kapodistrian University of Athens, Athens, Greece; ^3^Division of Botany, Department of Biology, National and Kapodistrian University of Athens, Athens, Greece

**Keywords:** actinobacteria, bioactive metabolites, skin aging, elastase inhibition, tyrosinase inhibition, antioxidant activity, autophagy activation, ubiquitin-proteasome system

## Abstract

Actinobacteria are a rich secondary metabolite source, accounting for nearly half of known bioactive microbial compounds, thus representing promising targets for novel bioactive molecule discovery. To explore potential antiaging compounds, we screened extracts from 980 actinobacterial strains isolated from diverse Greek ecosystems. Extracts were evaluated for elastase and tyrosinase inhibition *in vitro*, followed by toxicity and efficacy assessments in human cell lines. One *Amycolatopsis* and two *Streptomyces* strains exhibited significant tyrosinase inhibition, and one showed elastase inhibition, prompting further investigation. Culture optimization and fractionation of one of the most promising *Streptomyces* extracts resulted in the isolation of the six most bioactive and least toxic molecules, namely, Cyclo (L-proline-L-tyrosine) (1), Cyclo (Pro-Phe) (2), Lumichrome (3), P-(acetylamino) benzoic acid (4), Daidzein (5), and Uracil (6). These were tested for elastase and tyrosinase inhibition as well as antioxidant activity, and the activation of the autophagy-lysosome and the ubiquitin-proteasome system in cell lines and in *Drosophila melanogaster*. Molecules (1) and (4) demonstrated moderate elastase inhibition, while molecules (2), (3), (5), and (6) reduced reactive oxygen species under certain conditions. None activated the proteasome but all increased lysosomal activity in cell lines. Molecules (1), (2), (4), (6) were selected for study on *Drosophila*. Molecules (1) and (2) increased the activity of proteasome and molecules (1), (2), (4) increased the activity of lysosomes. All four molecules triggered antioxidant responses in *Drosophila*. This study highlights the potential of Greek actinobacterial biodiversity as a valuable resource for developing novel antiaging compounds with significant therapeutic implications for skin aging.

## 1 Introduction

Actinobacteria are Gram-positive bacteria with high G-C DNA content that belong to the phylum Actinomycetota. The majority are free-living organisms that are widely distributed in both terrestrial and aquatic ecosystems. Many actinobacteria produce a mycelium and reproduce by sporulation (Barka et al., 2016). *Streptomyces* is an especially important genus with over 800 species (Quinn et al., 2020) that has a global distribution and is abundant in soil, where it plays a key role in recycling cell walls of fungi and plants (Chater, 2016). Actinobacteria are especially important to humans because they produce a huge number of secondary metabolites. One review determined that from 33,500 bioactive microbial metabolites, 13,700 are derived from actinobacteria and 10,400 of those from the genus *Streptomyces* (Bérdy, 2012). It is estimated that every *Streptomyces* strain has the potential to produce more than 30 secondary metabolites on average (Lee et al., 2020). This very large number of secondary metabolites are likely produced due to the complexity of soil environments and the interactions of *Streptomyces* with other organisms over the course of long evolutionary periods (Seipke et al., 2012).

Actinobacterial bioactive compounds have been used in a wide range of applications, including antibiotics, biopesticides, plant growth regulators, antitumor and antiviral agents, pharmacological compounds, pigments, enzyme inhibitors, anti-inflammatory agents, single-cell protein feed, and biosurfactants, among others (Selim et al., 2021). This diversity and high number of useful secondary metabolites makes actinobacteria an ideal group to investigate for novel biomolecules. In addition, the geomorphological and climate conditions of Greece—such as its geographical position and dry Mediterranean climate - create soil reservoirs with high taxonomic and functional diversity of actinobacterial populations, offering a rich source of strains with potential pharmaceutical value (Laskaris and Karagouni, 2021).

The global population is aging, and it has been estimated that the elderly will constitute about one-fifth of the global population by 2050, which will result in a massive increase in aging associated diseases (Thomas and Rees, 2010). Aging consists of progressive physiological changes that alter normal biological functions, such as the ability to manage metabolic stress, and eventually lead to cellular senescence. Aging is characterized by genomic instability, telomere attrition, epigenetic alterations, loss of proteostasis, deregulated nutrient sensing, mitochondrial dysfunction, cellular senescence, stem cell exhaustion, and altered intercellular communication (Mishra et al., 2022). The most visible signs of aging are those affecting human skin. Aging results in cumulative changes in skin structure, function and appearance, including increased wrinkles, laxity, elastosis (accumulation of abnormal elastin), telangiectasia (spider veins), and aberrant pigmentation (senile lentigo or age spots) (Gu et al., 2020). Skin aging is the result of both endogenous and exogenous factors. Endogenous factors include the passage of time, genetic predispositions and the body’s hormonal balance (Paterska et al., 2024). The principal exogenous factors are sunlight (photoaging), air pollution, tobacco smoke, unhealthy eating, exposure to extreme temperatures, stress and lack of sleep (He et al., 2024). The contribution of these factors varies depending on the skin area; it is estimated that over 80% of facial skin aging is due to photoaging (Friedman, 2005).

One visible result of aging are wrinkles, which result from a decreased production of collagen and elastin, proteins that are vital for skin strength and resilience. Elastin plays a crucial role in skin elasticity, as it returns the skin to its normal configuration after being stretched or deformed (Shin et al., 2019). The decline in skin elasticity is mostly due to the reduction in the production of collagen and elastin (Alves et al., 2024). This is compounded by photoaging, which causes the degradation of elastic fibers of upper dermis by elastolytic enzymes, including matrix metalloproteinases and neutrophil elastases (Shin et al., 2023). Human skin fibroblast elastase induced by UV radiation plays a central role in wrinkle formation (Morisaki et al., 2010). However, inhibition of elastases has been shown to reduce wrinkle formation in experimental models (Tsuji et al., 2001).

Another sign of aging is senile lentigo, which most commonly occurs on exposed skin areas and is characterized by variably sized light- to dark-brownish macules and patches. Senescent fibroblasts from photoaged skin express high levels of pro-melanogenic growth factors, which activate melanocytes and result in localized hyperpigmentation (Kim et al., 2022). Because of that there is a high demand for inhibitors of melanogenesis that can help treat this hyperpigmentation disorder (Krambeck et al., 2023). Tyrosinase is a key enzyme involved in melanin biosynthesis and inhibition of its activity can thus be used for skin whitening treatments. As a result, there is a desire for bioactive compounds with anti-tyrosinase activity in the cosmetic industry (Esposito et al., 2021).

To understand these phenotypic aspects of aging, it is crucial to unravel the cellular processes that drive this phenomenon. Loss of proteostasis and increased Reactive Oxygen Species (ROS), are major characteristics of cellular aging. The ubiquitin-proteasome system is responsible for the proteolysis of damaged proteins, which is required for cellular homeostasis. Aging decreases proteasome activity and reduces the capability of cells to remove oxidatively modified proteins, which can lead to disease development (Löw, 2011). Activation of the proteasome via secondary metabolites has been demonstrated to extend the lifespan of human cell lines, marking it as a potential anti-aging treatment (Chondrogianni and Gonos, 2008). In addition, tyrosinase is degraded by the ubiquitin proteasome system. Compounds that upregulate tyrosinase degradation may therefore be effective as skin lightening cosmetics (Ando et al., 2009).

Another key-mechanism of cellular proteostasis is autophagy. Autophagy is a cellular process that recognizes, sequesters, and delivers cytosolic cargo to lysosomes for degradation (He and Klionsky, 2009). This process has been shown to decrease with aging and likely contributes to the development of age-related diseases, such as neurodegeneration and metabolic defects (Martinez-Lopez et al., 2015). In addition, autophagy may be able to delay the skin aging caused by ultraviolet radiation, meaning that its upregulation could help delay it (Wang et al., 2019).

Additionally, free radicals such as ROS are constantly produced by cellular processes. The decline of the innate antioxidant response during aging can lead to an increase in oxidative stress, which may be the cause of various age-related pathologies such as cancer (Sesti et al., 2012). In addition, free radicals can be generated by sun exposure and pollution that can degrade and impair the production of collagen which accelerates skin aging (Barnawi and Barnawi, 2021). Redox imbalance is mitigated by an intricate network of molecules that are regulated by transcription factors, which can quickly respond to changes in the oxidative state of the cell. Nuclear factor erythroid 2-related factor 2 (Nrf2) is a key modulator of antioxidant response in human cells, inducing the expression of several antioxidant proteins (Venugopal and Jaiswal, 1998). Nrf2 downregulation is associated with aging and age-related diseases (Sykiotis and Bohmann, 2010). The Nrf2 signaling is conducted through the recognition of an upstream sequence, known as Antioxidant Response Element (ARE). Mild activation of the *Drosophila’s* ortholog of Nrf2, named cap ‘n’ collar isoform C (CncC), results in lifespan extension and aging phenotype amelioration (Tsakiri et al., 2019). The mechanisms responsible for maintaining the appropriate oxidative equilibrium in the cell have been the topic of multiple studies, with efforts in finding new natural products that target those pathways (Sklirou et al., 2018).

There is considerable interest in the development of cosmeceuticals that can be used to combat skin aging (Alves et al., 2024). Cosmeceuticals are topical products that combine features of cosmetics, beautifying or enhancing appearance, with drugs, which therapeutically improve the skin’s physiology (Milam and Rieder, 2016). The global size of the cosmetics industry was reported at 380.2 billion in United States Dollars during 2019 and is projected to reach USD 463.5 billion by 2027 (Costa et al., 2022). There is thus great commercial and public interest in the discovery of novel active secondary metabolites that can be used as the active compounds of cosmeceuticals with antiaging activity. Numerous bioactive compounds with antiaging activity have been isolated from plants and fungi (Martel et al., 2019), but actinobacteria are also of great interest. Representative compounds include the tyrosinase inhibitors trichostatin A and trichostatic acid B from *Streptomyces* sp. CA-129531; streptochlorin (SF-2583A; 12815A) and 12815B from *S. roseolilacinus* NBRC 12815; lorneic acids E–J from endophytic *Streptomyces* sp. KIB-H1289; and undecylprodigiosin from a marine *Streptomyces* sp. SNA-077; additional reports describe extract-level or unnamed tyrosinase inhibitors from *Streptomyces* spp. (Anzai et al., 2008; Bang et al., 2008; Chang et al., 2008; Ashok et al., 2023; Dahal et al., 2017, 2020; reviewed by Fernandes and Kerkar, 2019). For elastase, examples include the actinomycete-derived elastatinal, elasnin from *S. noboritoensis*, osmanicin from *S. osmaniensis*, and the diketopiperazine cyclo(S-Phe-S-Pro) “SMFEI02” from *S. lavendulae* SMF11; extract-level inhibition is also reported from *Micromonospora fluostatini* SH-82 *Streptomyces* sp. G-18 and other actinobacteria (Hassane et al., 2020; Ashok et al., 2023; Dahal et al., 2017, 2020). This indicates that actinobacteria are a promising potential source of compounds with anti-aging activity.

The objective of this project was therefore to discover novel active compounds with anti-wrinkle or skin whitening activity derived from Greek actinobacteria, as well as to test identified compounds for more broad antiaging activity by examining their effects on autophagy and the proteasome system, since these pathways could also help ameliorate skin aging.

## 2 Materials and methods

### 2.1 General experimental procedures

High-Resolution Electrospray Ionization Mass Spectrometry (HRESIMS) data were acquired with a hybrid ESI-LTQ Orbitrap Discovery XL mass spectrometer (Thermo Scientific, Germany). The identification of the compounds was performed on a Hypersil Gold UPLC C18, column (2.1 × 150 mm, 1.9 μm; Thermo Fisher Scientific) using a mobile phase consisting of H_2_O with 0.1% (v/v) formic acid (A) and methanol (B). A 16 min gradient method was applied at the flow rate of 0.26 mL/min. The gradient elution started with 5% of B reaching 95% in 11 min. A 3 min washing step followed using 95% of B before returning, in 0.1 min, to the initial conditions for equilibration until the end of the 16 min gradient method. The column temperature was kept at 40 °C while the sample tray temperature was set at 4 °C. A full scan with a mass range of 120–1,200 Da on a centroid mode was applied while HRMS data (70,000 resolution) were recorded in both negative and positive ionization modes. 1D and 2D NMR spectra were obtained with a Bruker^®^ Avance III 600 MHz and Bruker® AvanceNEO 400 MHz spectrometers. The HPLC system consisted of an ECOM^®^ ECP2010 pump, an ECOM^®^ ECB2004 gradient box and degasser, a MISTRAL^®^ column oven (Spark Holland, Emmen, Holland), an ALIAS^®^ generic autosampler (Spark Holland, Emmen, Holland), an ECOM® ECDA2800 Diode Array Detector (Prague, Czech Republic), a SOFTA^®^ model 300S ELSD (Teledyne Isco, Nebraska, USA) and an ECOM^®^ ECF2000 Fraction Collector. The analytical column used was a Supelco^®^ 5 microns 25 cm x 4.6 mm C18 and the semi-preparative column was a Fortis^®^ 5 microns 250 cm x 10 mm C18. Chromatography column had a diameter of 2.8 cm, pore size 2 and was undertaken using Sephadex^®^ LH-20.

### 2.2 Isolation of actinobacteria from soil

A modified version of a protocol previously employed by the authors was used (Katsifas et al., 1999). Sampling mainly took place from a variety of soils and rizospheres within Greece according to local legal regulations, though there were also some samples taken from leaves and marine sediments. A 1 g soil, root or leaf sample was placed in a 50 mL Falcon tube and 10 mL of Ringer’s solution (¼ strength) was added. The tubes were shaken for 1 h at 180 rpm and 30 °C. The supernatant was serially diluted in Ringer’s solution (¼ strength) and 100 μL of each dilution from 10^0^ to 10^−6^ were streaked in duplicate on Petri dishes containing Arginine Glycerol Salt medium (AGS) (Herron and Wellington, 1990), supplemented with 100 mg/L K_2_Cr_2_O_7_ as antifungal agent. The plates were incubated at 30 °C for 3–5 days. Colonies were selected for isolation based on their morphology, which had to be similar to that of streptomycetes, and recultivated until the cultures were confirmed to be axenic. The bacteria were stored at −80 °C as spore suspensions in 20% (v/v) glycerol solution.

### 2.3 Strain identification

Genomic DNA was isolated from three-day old cultures grown in Nutrient Broth at 30 °C, following the Joint Genome Institute’s protocol for bacterial genomic DNA isolation using the CTAB method.^1^ The isolated DNA was then used to amplify the 16S rRNA gene with Taq DNA Polymerase, employing the forward primer 27F (5′-AGAGTTTGATCCTGGCTCAG3′) and the reverse primer 1492R (5′TACGGTTACCTTGTTACGACTT-3′). The PCR reaction protocol consisted of 95 °C for 3 min, (95 °C for 30 s, 55 °C for 30 s, 72 °C for 2 min) x 30 cycles, and 72 °C for 10 min. The PCR products underwent gel electrophoresis and were then extracted and purified from agarose gel using the Macherey-Nagel Gel Extraction kit according to the manufacturer’s instructions. The 27F and 1492R primers were used for Sanger sequencing of the purified PCR product. The obtained sequences were compared with the GenBank 16S ribosomal RNA database using the BLASTn algorithm.^2^

### 2.4 Culture medium selection

A literature search between 2017 and 2022 found 108 publications that included 75 different nutrient substrates used for the isolation of secondary metabolites (Supplementary Table 2). The liquid media ISP1 (Tryptone Yeast Extract), ISP2 (Yeast Malt Extract) and ISP4 (Inorganic Salt Starch) (Atlas, 2010) were selected to be tested because they were the most used liquid media in our literature search. In addition, AGS (Arginine Glycerol Salt) (El-Nakeeb and Lechevalier, 1963) was chosen as it is the medium most often used in our laboratory for growing actinobacteria.

For the optimization of antiaging compound production, the selected strains were grown in the media AGS, ISP2, ISP4, and ISP3 (Oatmeal). ISP3 replaced ISP1 because the former medium’s components are more varied compared to the other media used and might lead to differences in bioactive molecule production in the studied bacteria.

### 2.5 Extraction

The following procedure was employed to generate cultures to undergo extraction: 10 μL of 20% glycerol spore suspension were added to 1 mL TSB (Tryptic Soy Broth) in a 2 mL Eppendorf tube. The pre-culture was grown at 30 °C for 3 days at 180 rpm. 500 μL of pre-culture was added to 10 mL of ISP4 liquid culture in 50 mL Falcon tubes and incubated at 30 °C for 7 days at 180 rpm.

An acetone-based extraction protocol was developed by combining equal volumes (10 mL) of liquid culture and acetone, followed by 1 min of vortexing and 45 min of ultrasonic treatment at 25 °C. The mixture was heated to 40 °C in a Reacti-Thermo III Heating and Stirring Module (Thermo Scientific) and the acetone was removed with a Reacti-Vap III Evaporator (Thermo Scientific). This was followed by a liquid–liquid extraction with an equivalent volume of EtOAc (2 cycles of 10 mL); 10 mL of EtOAc was added to 10 mL of lysed cells, followed by vortexing for 1 min, extraction in an ultrasonic bath for 45 min at 25 °C, and centrifugation for 15 min at 4 °C. The EtOAc extract was removed from the Falcon tube and evaporated under vacuum. The crude extract was dissolved in DMSO and frozen. 10 mL of MeOH was added to the remaining aqueous supernatant, followed by vortexing for 1 min, extraction in an ultrasonic bath for 45 min at 25 °C, and centrifugation for 15 min at 4 °C. 1.5 mL of hydroalcoholic extract was removed and evaporated under vacuum into an RVC 2–33 CDplus Rotary Vacuum Concentrator (Martin Christ). The crude extract was dissolved in DMSO and frozen. The four most active microbial strains, belonging to the *Amycolatopsis* and *Streptomyces* genera, were subjected to a scale up process (2 L) and the cultures were extracted using the same extraction protocol. The cultivation broth was then extracted two times using 2 L of EtOAc in a 6 L separation funnel. The EtOAc phase was collected in round flasks and sodium sulfate anhydrous was added to bind the remaining water. The extracts were filtered using filter paper and ethyl acetate was removed using RotaVapor R-300, Buchi^®^. Dry extracts were stored in penicillin vials of 8 mL at −20 °C. The above extraction workflow was developed *de novo* for this study and was not adapted from previously published protocols. All parameters (solvent volumes/ratios, ultrasonication time/temperature, centrifugation conditions, and extraction order) were empirically optimized. Routine steps (e.g., drying organic phases with anhydrous sodium sulfate and solvent removal by rotary evaporation) followed the manufacturers’ instructions for the instruments listed.

### 2.6 Fractionation and isolation

A fractionation and isolation protocol was developed to achieve purification of the compounds. High Performance Liquid Chromatography was used for fractionation, recording PDA from 200 to 800 nm. The mobile phases were 0.1% TFA aqueous solution (A) and acetonitrile (B) with a gradient: 0–10 min, 5% B; 10–60 min, 5–100% B; 60–72 min, 100% B; 72–82 min, 5% B. The flow rate was 1.00 mL min^−1^ and 1.50 mL min^−1^ and the injection volumes were 20 μL and 60–220 μL for analytical and semi-preparative experiments, respectively. Extracts (20 mg/mL in methanol) were fractionated into 48 fractions (1.5 min per fraction). The EtOAc extract of *Streptomyces* sp. ATHUBA 292 demonstrated the highest tyrosinase inhibition rate in cell-free assays during the initial screening (87.6438%) and was thus selected for scaled-up cultivation. The strain was cultivated in 2 L, extracted with EtOAc, and further fractionated using Sephadex^®^ LH-20 chromatography (2.8 cm diameter, pore size 2) with CHCl₃/MeOH (66:33 v/v) as mobile phase, yielding 70 fractions combined into 14. Additional fractionation used Sephadex^®^ LH-20 under gradient conditions (CHCl₃/MeOH: 80:20, 66:33, 50:50), producing 100 fractions combined into 14. Thin-layer chromatography (TLC) was employed for compound screening and fraction pooling, using silica gel 60 F254 plates (20 × 20 cm), visualized at 254 and 366 nm after vanillin/sulfuric acid staining. DCM/MeOH (95:5 v/v) served as the mobile phase. Compounds of interest were recovered by scraping, eluting with ethyl acetate, drying, and storing at −20 °C.

### 2.7 *In vitro* enzymatic inhibition of elastase and tyrosinase

The elastase inhibitory activity of the extracts was evaluated using elastase from porcine pancreas (PPE) type IV (Merck KGaA, Darmstadt, Germany) and the substrate N-succinyl-Ala-Ala-Ala-p-nitroanilide (Merck KGaA, Darmstadt, Germany), as previously described (Hassane et al., 2020; Samy et al., 2021). More specifically, in a 96 well plate 10 μL of each extract (200 μg/mL) was incubated for 15 min, in room temperature, avoiding light exposure, with 70 μL of Trizma-Base buffer (50 mM, pH 7,5) and 5 μL PPE (0,4,725 U/mL). After addition of 15 μL of substrate (0.903 mg/mL) and incubation in 37 °C for 30 min in the dark, the absorbance of the produced p-nitroaniline was measured at 405 nm, using a spark multimode microplate reader (Tecan). Elastatinal (12.5 μg/mL) was used as positive control and the extract was substituted with Trizma buffer to generate the experiment’s control. For each extract a different blank was generated with the combination of extract (or buffer for the control), buffer and substrate but not PPE. The capacity of the extracts to inhibit the catalytic action of tyrosinase in the oxidation of L-DOPA to dopachrome was determined, measuring the absorbance at 475 nm as previously reported (Campos et al., 2020; Samy et al., 2021). In detail the protocol starts with a 10-min incubation, in room temperature, in the dark, of 40 μL PBS (Phosphate Buffer Saline, pH 6.8), 20 μL of extract (200 μg/mL) and 20 μL of tyrosinase enzyme (Sigma Aldrich) at final concentration of 92 U/mL, loaded in a 96 well plate. Afterwards 20 μL of L-DOPA (2.5 mM) are added and the plate is incubated for 5 min at room temperature, in the dark. Kojic acid (2 μg/mL) was used as a positive control, mixture with all the components except the enzyme was used as blank (different for each sample and the control) and a mixture where the extract was substituted with buffer was used as the experiment’s control. The mathematical equation used to calculate the percentage of inhibition for both enzymes is the following:


[(A−B)−(C−D)]÷(A−B)×100

(where A and B are the absorbances of the experiment’s control and control’s blank respectively, C is the absorbance of the sample and D is the absorbance measured for each sample blank). A concentration of 200 μg/mL of each extract was used in order to ensure sufficient interaction between the extract components and the enzymes, allowing us to observe potential inhibitory effects.

### 2.8 Cell lines and cell culture conditions

Human foreskin fibroblasts (BJ), mouse skin melanoma cells (B16-F10) and human immortalized keratinocytes (HaCaT), were obtained from the American Tissue Culture Collection (ATCC). All cell lines were cultured in Dulbecco’s Modified Eagle’s Medium (DMEM) (Thermo Fisher Scientific Inc., Waltham, MA, USA) supplemented with 10% (v/v) fetal bovine serum (FBS) and 1% (v/v) non-essential amino acids and were maintained in conditions of 5% CO_2_, 95% humidity and 37 °C. In all experimental procedures applied, cells were subcultured by using a trypsin/EDTA solution (Thermo Fisher Scientific Inc., Waltham, MA, USA).

### 2.9 Cell-based anti-tyrosinase activity

Tyrosinase activity in cells was measured using the oxidation of L-DOPA. B16-F10 cells were plated in 6-well plates and after 24 h were incubated with 50 μg/mL of each extract for 24 h. Cells were then lysed with phosphate-buffered saline (pH 6.8) containing 1% Triton X-100, and after the protein concentration was determined, 30 μg of protein were incubated with 5 mM L-DOPA (Sigma-Aldrich) at room temperature for 10 min in the dark. The absorbance at 475 nm was measured using Spark multimode microplate reader (Tecan life sciences). Kojic acid was used as positive control as previously described (Samy et al., 2021). The concentration of 50 μg/mL was chosen to avoid cytotoxicity and better reflect physiologically relevant conditions.

### 2.10 Cell-based anti-elastase activity

Elastase activity in cells was measured using the amount of released p-nitroaniline, which was hydrolyzed from the substrate (N-succinyl-Ala-Ala-Ala-p-nitroanilide). BJ fibroblasts were seeded into 6-well plates and after 24 h were treated with 50 μg/mL of the extracts for 24 h. Cells were then lysed in 100 mM Tris–HCl (pH 7.6) with 0.1% Triton X-100 buffer and subsequently, 2 mM N-Succinyl-Ala-Ala-Ala-p-nitroanilide (Sigma-Aldrich) was added to each well, followed by incubation at 37 °C for 1 h. The absorbance at 405 nm was measured using Spark multimode microplate reader (Tecan life sciences). Protease inhibitor (Sigma-Aldrich) was used as positive control. This protocol is followed as previously described (Samy et al., 2021).

### 2.11 Cell viability assay

Cells were plated in flat-bottomed 96-well microplates and after 24 h were incubated with different concentrations of extracts for 24 h. The medium was then replaced by 3-(4,5-dimethylthiazol-2-yl)-2,5-diphenyltetrazolium bromide (MTT, Sigma-Aldrich) dissolved at a final concentration of 1 mg/mL in serum-free, phenol red-free medium. The formazan crystals that formed then dissolved in isopropanol and the solution absorbance was measured at 570 nm wavelength using spark multimode microplate reader (Tecan). The survival of control cells, treated with an equal amount of the sample’s solvent, was arbitrarily set to 100%. The viability of each sample was calculated relative to control, using the measured absorbances in the following equation:


(sample−blank÷control−blank)×100

Unseeded wells, where all the procedure was followed as in the samples, were used as blank. This protocol is also previously described elsewhere (Samy et al., 2021).

### 2.12 Testing of different culture media for effects on elastase and tyrosinase inhibitor production

This protocol was developed by the authors to examine the effects of culture media on bioactive molecule production. 50 μL of spore suspension from strains that produced extracts with antiaging effects were added to 50 mL TSB in a 100 mL flask. The pre-culture grew at 30 °C for 3 days at 180 rpm. 3 mL of pre-culture were added to 150 mL of nutrient within a 500 mL flask. Four liquid media (AGS, ISP2, ISP3, ISP4) were used in triplicate. The 12 flasks were incubated at 30 °C for 7 days at 180 rpm. The extraction protocol was the same as in the liquid culture metabolite extraction section, but the reagent volumes were scaled up for 150 mL of culture.

### 2.13 Culture scale-up

The scale-up methodology was developed by the authors. 50 μL of spore suspension were added to four 100 mL flasks containing 50 mL TSB. The pre-culture grew at 30 °C for 3 days at 180 rpm. Two 5 L flasks had 4 L of ISP4 culture added to them and were inoculated with 80 mL of preculture. The cultures were incubated at 30 °C for 9 days in order to allow secondary metabolites to accumulate. A magnetic stirrer set at 100 rpm together with an air pump connected to silicon tubes immersed in the cultures were used to oxygenate the large-scale cultures. A Balston microfiber filter and 0.2 μm syringe filters were used to prevent contamination via the air supply. The 8 L of culture were stored at −20 °C until used.

### 2.14 Measurement of reactive oxygen species (ROS)

To determine ROS levels, cultured cells were collected in PBS and incubated in CM-H_2_DCFDA dye (Invitrogen, Carlsbad, CA, USA) for 30 min at 25 °C in the dark. Following centrifugation and dye removal, cells were incubated in PBS for 10 min at 24 °C to allow cellular esterases to hydrolyze the acetoxymethyl ester or acetate groups and render the dye responsive to oxidation. Samples were washed in PBS, lysed in Nonidet P-40 lysis buffer (1% Nonidet P-40, 150 mM NaCl, and 50 mM Tris, pH 8.0), and cleared by centrifugation at 19,000 × g for 10 min at 4 °C. The supernatant was diluted 1:4 (v/v) in ddH_2_O, and fluorescent dichlorodihydrofluorescein was measured using a Spark multimode microplate reader (Tecan life sciences), at excitation 490 nm/emission 540 nm. Negative controls were either unstained cells incubated with PBS buffer alone to detect autofluorescence or cell-free mixtures of dye and buffers. This protocol was adjusted from the manual provided by Invitrogen for CM-H_2_DCFDA dye also described elsewhere (Louka et al., 2021).

### 2.15 Measurement of proteasome proteolytic activity

Cells or isolated flies’ somatic tissues were lysed on ice using buffers suitable for the isolation of 26S proteasomes (0.2% Nonidet P-40, 5 mM ATP, 10% glycerol, 20 mM KCl, 1 mM EDTA, 1 mM dithiothreitol and 20 mM Tris, pH 7.6). Lysates were cleared by centrifugation at 19,000 × g (4 °C) and 5 μg of proteins, measured by Bradford assay (BioRad) (Kielkopf et al., 2020), were immediately used to determine the chymotrypsin-like (CT-L), and caspase-like (CL) activities of the proteasome. CT-L activity was measured by recording the hydrolysis of the fluorogenic peptide Suc-Leu-Leu-Val-Tyr-AMC (Suc-LLVY-AMC, Enzo Life Sciences) and CL activity was measured using Z-Leu-Leu-Glu-AMC (Z-LLE-AMC, Enzo Life Sciences). The isolated proteins were incubated with 50 μM of the appropriate fluorigenic peptides for 30 min at 37 °C, avoiding light exposure. Fluorescence was measured using a Spark multimode microplate reader (Tecan life sciences), at excitation and emission wavelengths of 380 and 460 nm, respectively. Fluorescence intensity was normalized to the total protein level per sample and expressed as the relative percentage vs. control (DMSO); in experiments using flies’ tissues equal numbers of male and female flies were used. Each sample was prepared in duplicate. This protocol was adjusted from the manual provided by the fluorogenic peptides’ manufacturer, and it is also described elsewhere (Louka et al., 2021).

### 2.16 Measurement of cathepsins’ enzymatic activities

Cells or isolated flies’ somatic tissues were lysed on ice in 1 mM dithiothreitol and 50 mM Tris, pH 4.0, and the lysates were cleared at 14,000 × g for 20 min at 4 °C. After the protein content was measured by Bradford assay (BioRad) (Kielkopf et al., 2020), 5 mg of protein were incubated in the reaction buffer (50 mM sodium acetate, 8 mM cysteine hydrochloride, 1 mM EDTA, pH 4.0) containing 500 μM of the substrate- Z-Phe-Arg-AMC (Z-FR-AMC, Enzo Life Sciences) for 30 min at 37 °C. The fluorescence was measured in a Spark multimode microplate reader (Tecan life sciences), at excitation and emission wavelengths of 380 and 460 nm, respectively. This protocol is also described elsewhere (Louka et al., 2021).

### 2.17 *In vivo* measurement of antioxidant response

Transgenic flies (GstD1-ARE: GFP/ II) that express the GstD1-GFP reporter (Glutathione S-transferase Delta 1 fused with Green Fluorescent Protein), regulated by an ARE, were cultured in supplemented food, with 1 μM and 10 μM of the isolated metabolites. After 7 days of culture, somatic tissues of each group were collected and homogenized in ice-cold Nonidet P-40 buffer. After centrifugation (19,000 × g, 4 °C, 10 min) the supernatant was collected, and the protein concentration was determined using Bradford assay (Kielkopf et al., 2020). The GFP levels were calculated as Relative Fluorescence Units per microgram of protein (RFUs/μg) by measuring GFP’s fluorescence in Spark multimode microplate reader (Tecan life sciences), at 395 nm excitation and 508 nm emission wavelengths. Flies that were supplemented with DMSO, which serves as a solvent for metabolites, were used as a control group. Female flies from the samples were used for visualization, by immobilizing their bodies on glass observation slides with Vaseline and capturing the fluorescence in images using Leica stereo microscope (M205 FA, Leica, DE). Wild-type flies that do not express GFP as well as transgenic flies not cultured with the selected metabolites were used as controls.

### 2.18 Statistical analysis

All the experiments were performed at least in duplicates. Statistical significance was evaluated with Student’s t-test and indicated with asterisks, two (**) for *p* < 0.01 and one (*) for *p* < 0.05. Data visualization was completed using Graphpad Prism 8 (version 8.0.2). All barplots represent the mean of each set of measurements and error bars are added to indicate the Standard Deviation (SD).

## 3 Results

Several Greek sites were selected known or suspected of harboring actinobacteria. Samples were taken from soil, plant rhizospheres, leaves, and marine sediments. From all the isolates, 980 strains were identified as actinobacteria based on morphology and 16S rRNA gene sequences. The strains used in this project include members of the genera *Streptomyces*, *Amycolatopsis*, *Nocardiopsis*, *Nocardia* and *Micromonospora*. The strains and their isolation sites are presented in Supplementary Table 1.

### 3.1 Creation of extract library

Out of 75 potential liquid media identified in our literature search (Supplementary Table 2), the following commonly used liquid media were tested: ISP1 (Tryptone Yeast Extract), ISP2 (Yeast Malt Extract), ISP4 (Inorganic Salt Starch) and AGS (Arginine Glycerol Salt). ISP4 was selected for use in the screening, as it generated adequate biomass, measured in dry weight, without introducing a large number of molecules from its constituents into the extracts, as determined by HPLC. ISP1 and ISP2 generated adequate biomass but also introduced large numbers of molecules in the extracts, while AGS did not produce adequate biomass (data not shown). Ethyl acetate and methanol extracts were then generated from 980 actinobacterial strains grown in ISP4, yielding a total of 1960 extracts.

### 3.2 Extract screening for *in vitro* antiaging activity

**Figure 1:**
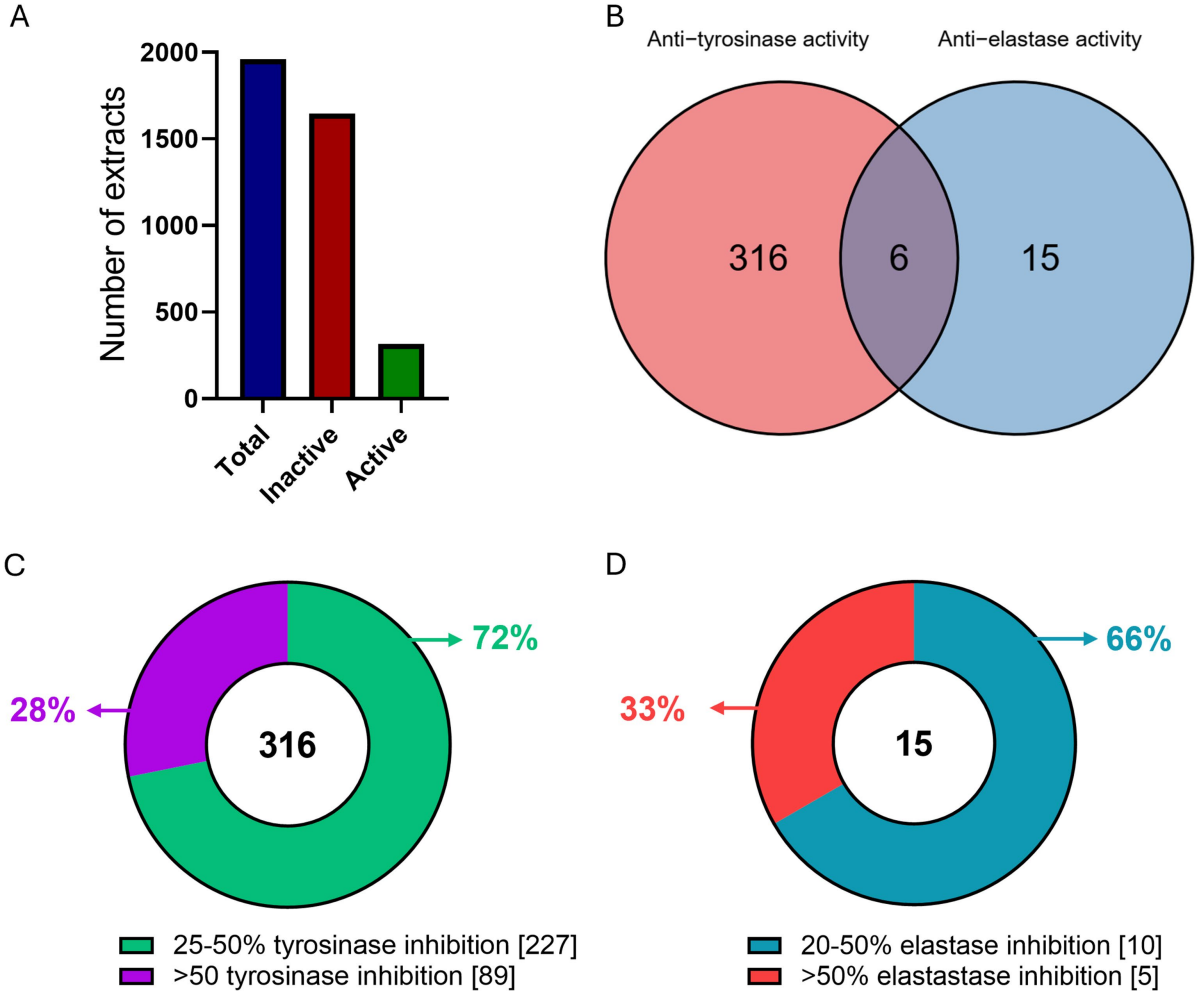
Overview of extract screening. **(A)** Number of total (blue), inactive (red) and active (green) extracts as tested in *in vitro* enzymatic assays. **(B)** Venn diagram of the tyrosinase and elastase active extracts. **(C)** Number of extracts per *in vitro* enzymatic inhibition rate for **(C)** tyrosinase, and **(D)** elastase.

The results of the tyrosinase and elastase enzymatic inhibition tests on 1960 extracts (200 μg/mL) revealed that 337 of them exhibited enzymatic inhibition potential at a rate of >25 and >20% in tyrosinase and/or elastase respectively, thus marked as active (Figure 1A). Specifically, of the 316 tyrosinase-only active extracts, 227 had 25–50% inhibition rate and the remaining 89 had over 50% inhibition activity potential (Figure 1C). Regarding elastase inhibition, we identified 15 elastase-only active extracts, 10 of which exhibited 20–50% elastase inhibition and the other 5 had over 50% elastase inhibition activity (Figure 1D). Furthermore, 6 extracts showed combined activity against both enzymes in *in vitro* assays (Figure 1B). Ethyl acetate extracts demonstrated higher inhibitory activity than methanol extracts: there were 200 active ethyl acetate extracts compared to 141 active methanol extracts. More specifically, 181 ethyl acetate extracts inhibited tyrosinase inhibition compared to 139 methanol extracts, and 19 ethyl acetate extracts were effective against elastase, compared to only 2 methanol extracts. Thus 18.5% of all ethyl acetate extracts showed anti-tyrosinase activity compared to 14.2% of all methanol extracts, which was significantly larger (*p* = 0.0103), and 1.9% of ethyl acetate extracts demonstrated anti-elastase activity compared to 0.2% of all methanol extracts, which was also significantly greater (*p* = 0.000192).

### 3.3 Cytotoxic and cell-based evaluation of the most active extracts

**Figure 2:**
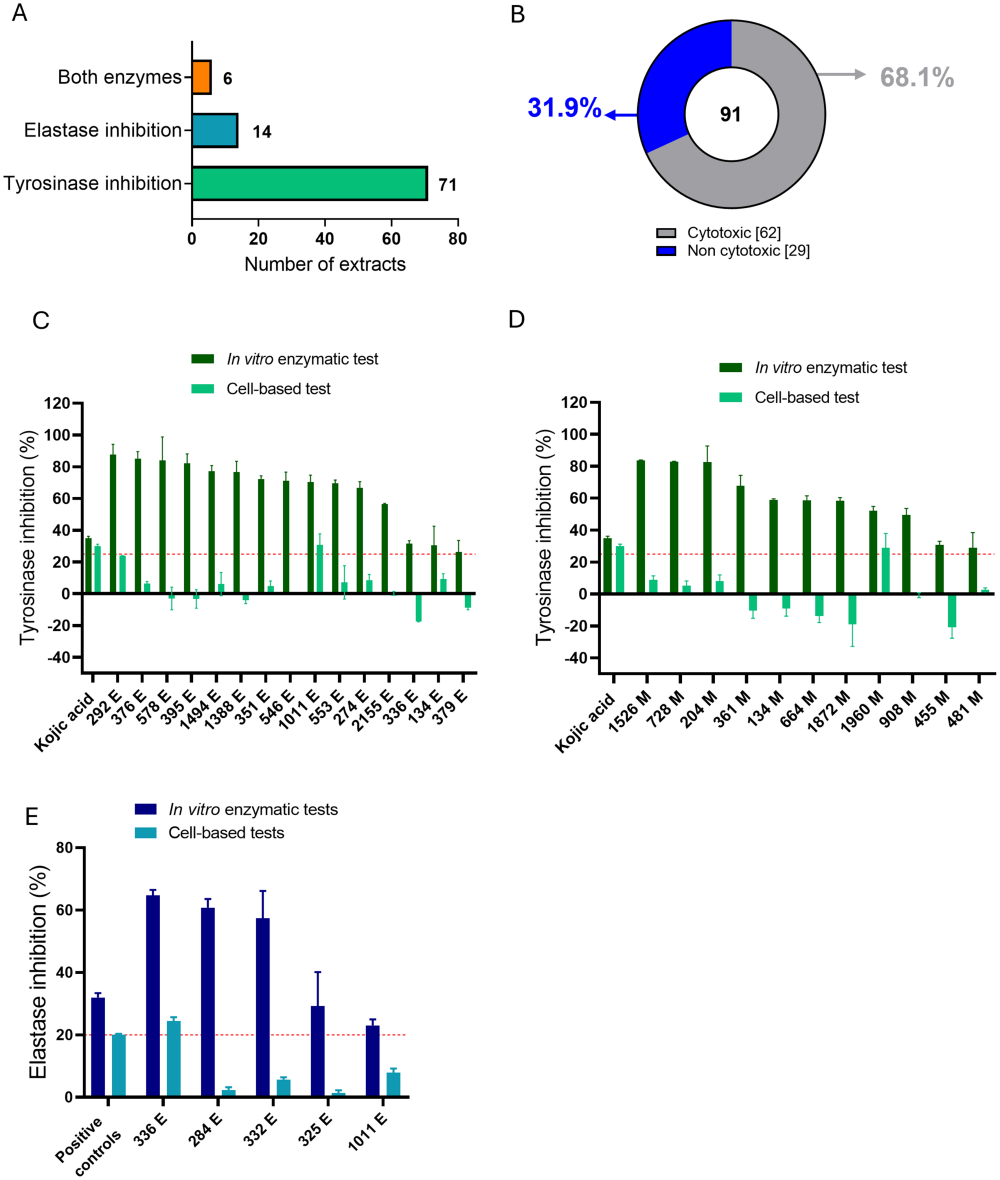
Overview of *in vitro* enzymatic and *in vitro* cell-based tests for the most active ATHUBA strain extracts. **(A)** Numbers of active extracts per enzymatic inhibition that were chosen for further tests. **(B)** Pie chart showing the percentages of the toxic and the non-toxic extracts as tested on BJ and/or HaCaT cells. **(C)** Bar plot of the 15 non-cytotoxic EtOAc extracts that exhibit tyrosinase inhibitory activity, **(D)** Bar plot of the 11 non-cytotoxic MeOH extracts that exhibit tyrosinase inhibitory activity. Dark green: Tyrosinase inhibition percentages for the *in vitro* enzymatic tests. Light green: Tyrosinase inhibitory percentages for the *in vitro* cell-based test on B16-F10 melanocytes. **(E)** Bar plot of the 5 non-cytotoxic extracts that exhibit elastase inhibitory activity. Dark blue: Elastase inhibition percentages of the *in vitro* enzymatic tests. Light blue: Elastase inhibitory percentages for the *in vitro* cell-based test on BJ cells. Two extracts (336 E and 1,011 E) are present in both **(C, D)**, since they presented initial inhibition in both enzymes in *in vitro* enzymatic tests. Kojic acid 2 μg/mL and 100 μg/mL was used as positive control for *in vitro* enzymatic and cell-based tyrosinase inhibition activity, respectively. Elastatinal (2 μg/mL) was used as a positive control for *in vitro* elastase inhibition activity and protease inhibitor (1X) was used as a positive control in the cell-based assay. Red dotted lines represent the inhibition limit of 25 and 20% for tyrosinase and elastase activity, respectively. Extract concentration: 200 μg/mL for *in vitro* enzymatic tests and 50 μg/mL for cell-based tests, ±SD.

We subsequently selected extracts with high inhibition rates in *in vitro* enzyme tests to assess their impact on cell viability in BJ and HaCaT cell lines at a concentration of 50 μg/mL. Of the 91 selected extracts (Supplementary Table 3), 71 of them had only anti-tyrosinase activity and 14 of them had only anti-elastase activity in *in vitro* enzymatic assays, while the remaining 6 had inhibition activity against both enzymes (Figure 2A). Cytotoxic evaluation on these 91 selected extracts, revealed that 62 of them were cytotoxic (reducing cell viability by more than 10% after 24 h) in one or both the cell lines tested, up to a concentration of 10 μg/mL. The remaining 29 extracts were marked as non-toxic in either of the cell lines (Figure 2B).

We continued the evaluation on the 29 non-cytotoxic extracts by testing their enzymatic inhibition ability in cell-based assays. The extracts were tested on B16-F10 melanocytes and/or BJ fibroblasts, for their anti-tyrosinase and/or anti-elastase activity, respectively. Cell-based assays were performed based on the inhibitory activity that was measured in enzymatic assays. A tyrosinase-inhibiting extract was thus only tested for its anti-tyrosinase activity on melanocytes, an elastase-inhibiting extract was only tested for its anti-elastase activity in fibroblasts, and those who showed inhibition against both enzymes in *in vitro* enzymatic assays, were tested for their ability to inhibit both the enzymes in cell-based assays accordingly. Of the 29 non-cytotoxic extracts that were evaluated, 24 were active only against tyrosinase, 3 only against elastase and 2 against both enzymes. Of the 26 anti-tyrosinase extracts, 2 EtOAc (marked as E) (ATHUBA 292 E and ATHUBA 1011 E) (Figure 2C) and 1 MeOH (marked as M) extract (ATHUBA 1960 M) (Figure 2D) showed high tyrosinase inhibitory activity (~25%) in melanocytes. In addition, only 1 (ATHUBA 336 *Ε*) out of the 5 non-cytotoxic anti-elastase extracts exhibited high elastase inhibitory activity (>20%) in BJ fibroblasts (Figure 2E).

ATHUBA 292 (16S ribosomal RNA gene Genbank accession number PV793531), ATHUBA 336 (PV793532) and ATHUBA 1011 (PV793534) belonged to the genus *Streptomyces*, while ATHUBA 1011 (PV793533) belonged to the genus *Amycolatopsis*.

### 3.4 Testing of different culture media for effects on elastase and tyrosinase inhibitor production

**Figure 3:**
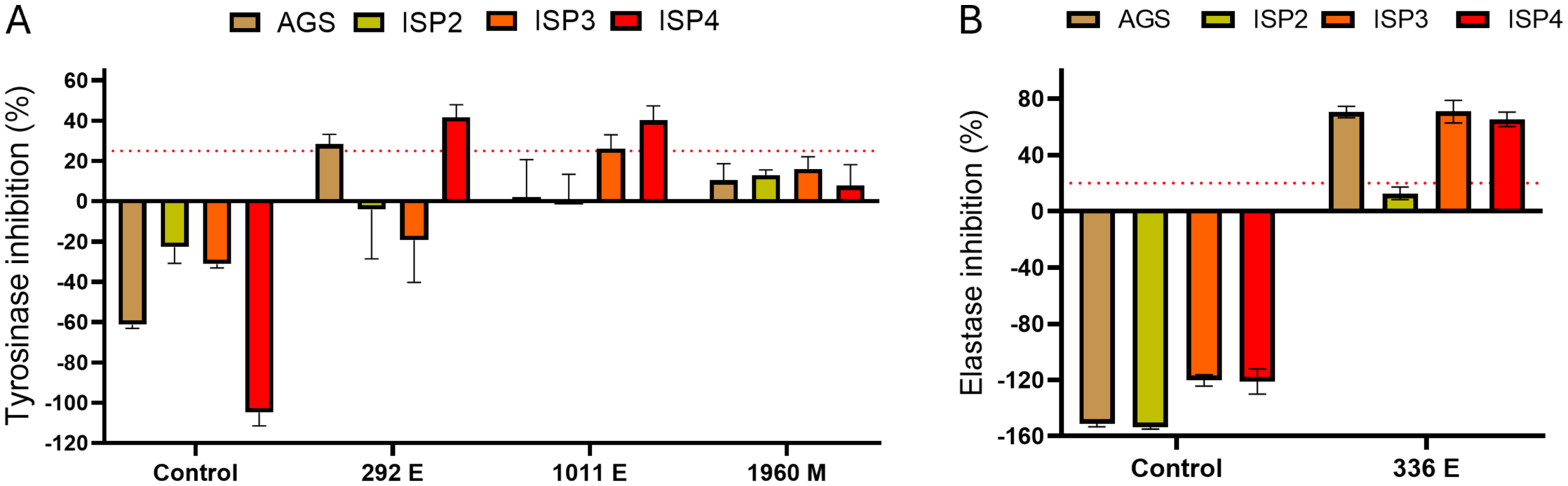
Culture medium tests for optimum elastase and tyrosinase inhibition. Culture mediums AGS, ISP2, ISP3, and ISP4 tested for optimum tyrosinase and elastase inhibition. Extracts ATHUBA 292 E, 1011 E, and 1960 M used for *in vitro* enzymatic tyrosinase inhibition tests **(A)**, and ATHUBA 336 E for *in vitro* elastase inhibition tests **(B)**. Extract from medium not used to grow bacteria was used as a control. Red dotted lines represent the inhibition limit of 25 and 20% for tyrosinase and elastase activity, respectively. Extract concentration: 200 μg/mL, ±SD.

To further test and improve the capability of the selected strains to produce metabolites with anti-tyrosinase and/or anti-elastase activity, we performed *in vitro* enzymatic assays from extracts of cultures grown in 4 different media in order to determine whether other media improved the production of these inhibitors (Figure 3). The extraction protocol used was the same as in the initial screening. The *Amycolatopsis* sp. ATHUBA 1011 E and *Streptomyces* sp. ATHUBA 292 E extracts showed the greatest tyrosinase inhibition when grown on ISP4 (Figure 3A). The *Streptomyces* sp. ATHUBA 1960 M extracts showed no significant tyrosinase inhibition on any media. The *Streptomyces* sp. ATHUBA 336 E extract showed similar elastase inhibition when the strain was grown on AGS, ISP3 and ISP4 (Figure 3B).

The extracts ATHUBA 292 E, ATHUBA 1011 E and ATHUBA 336 E were selected for further study. ATHUBA 1960 M showed no activity on any medium, indicating that production of the active metabolite in this strain is unstable. For all three strains, ISP4 was selected for further experiments as extracts from strains grown in this medium either showed greater inhibition or similar inhibition compared to other media.

### 3.5 Selection and fractionation of ATHUBA 292 extract

**Figure 4:**
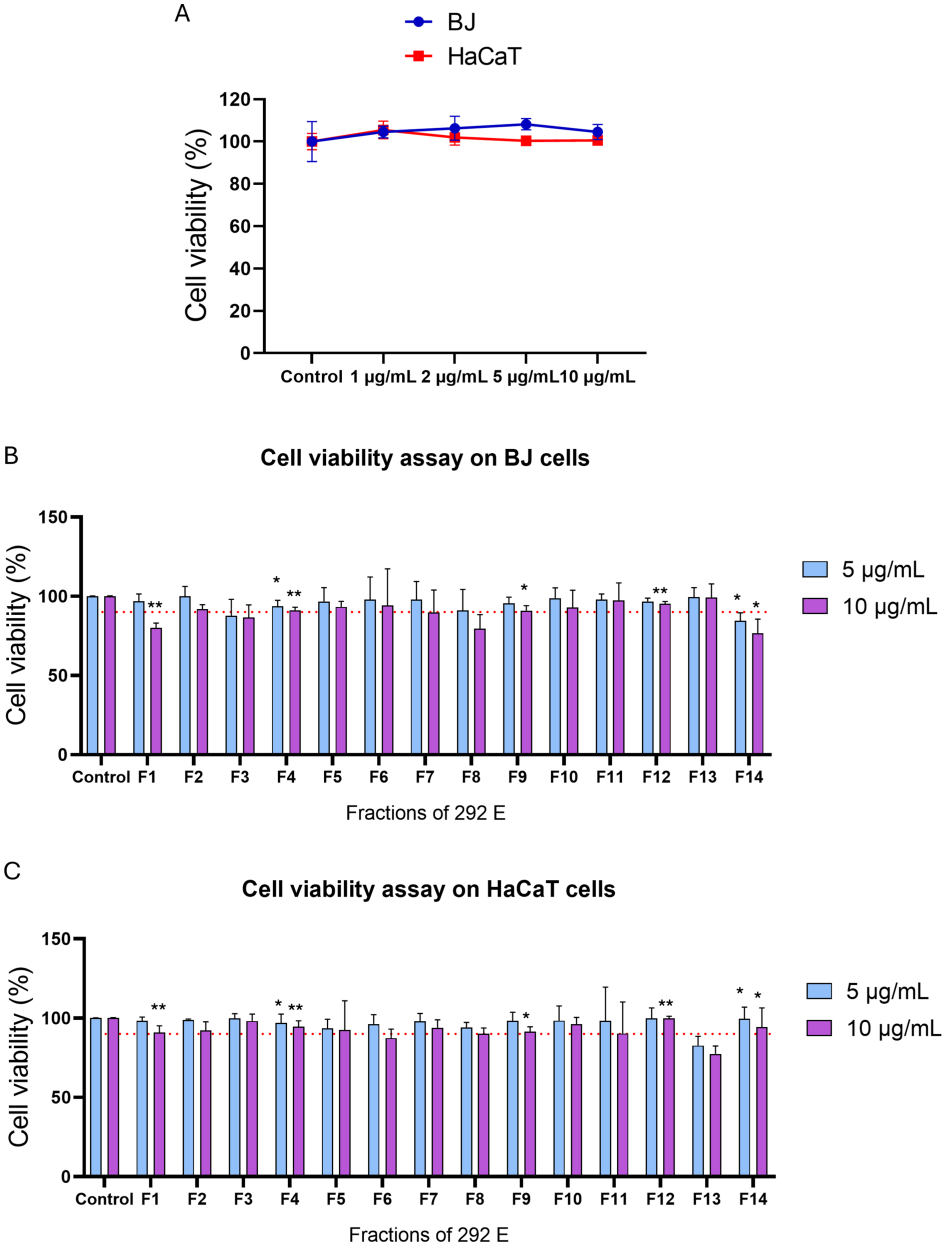
Cell viability assay of extract ATHUBA 292 E and its fractions. Cell viability (%) after 24 h of treatment of **(A)** ATHUBA 292 extract on BJ and HaCaT cells up to 10 μg/mL, and ATHUBA 292 E fractions on **(B)** BJ and **(C)** HaCaT cells. The red dotted lines represent the cell viability limit (90%). Control cells were treated with DMSO and were set to 100%, *p < 0.05, **p < 0.01, ±SD.

**Figure 5:**
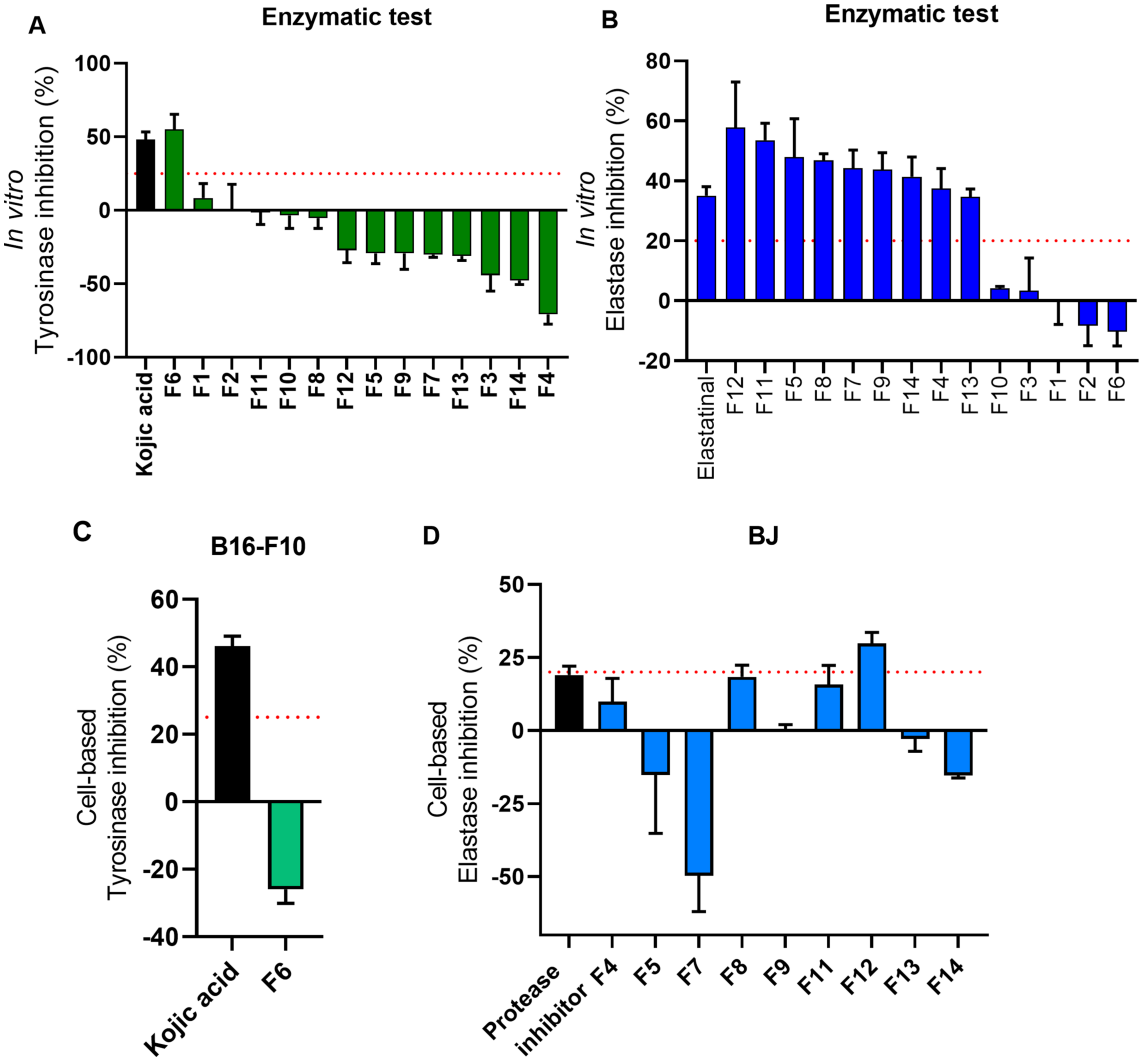
Overview of cell-based evaluations of ATHUBA 292 fractions. **(A)** *In vitro* tyrosinase inhibition rates of all 14 fractions (%). **(B)** *In vitro* elastase inhibition (%) of all 14 fractions. Kojic acid (2 μg/mL) and elastatinal (0.5 μg/mL) were used as positive controls for tyrosinase and elastase, respectively. Fraction concentration: 200 μg/mL, ±SD. **(C)** Tyrosinase inhibition (%) of fraction F6 in B16-F10 cells. Kojic acid (100 μg/mL) was used as positive control. **(D)** Elastase inhibition (%) in BJ cells. Protease inhibitor (1X) was used as positive control for elastase. The red dotted lines represent the inhibition limit of 25 and 20% for tyrosinase and elastase activity, respectively. Fraction concentration: 2 μg/mL, ±SD.

The selection of ATHUBA 292 E extract was based on its high inhibitory activity on both *in vitro* enzymatic and cell-based assays. Treatment with ATHUBA 292 E extract on BJ and HaCaT cells for 24 h showed no significant reduction on cell viability up to a 10 μg/mL concentration (Figure 4A) and we therefore proceeded with its fractionation. The fractionation process resulted in 14 fractions. All fractions were tested for their ability to inhibit elastase and/or tyrosinase in *in vitro* enzymatic and cell-based assays. Fraction F6 is the only one to have high (~55%) tyrosinase inhibition activity, while F4, F5, F7, F8, F9, F11, F12, F13, and F14 have anti-elastase activity (~37–57%). The rest of the fractions showed no activity (<25%) in *in vitro* enzymatic assays (Figures 5A,B). The active fractions were further tested on B16-F10 melanocytes and BJ fibroblasts for their possible intracellular activity. Fraction F6 did not confirm its anti-tyrosinase activity in melanocytes (Figure 5C). In fibroblasts, fraction F12 was the most active against elastase (~29% inhibition), and F8 and F11 followed with inhibition rates at 18 and 15% accordingly (Figure 5D). Next, all fractions were tested for their potential cyto-toxic effect on BJ and HaCaT cells. Most fractions were cytotoxic to either BJ or HaCaT cells. Specifically, fractions F1, F3, F8, and F14 were toxic to BJ cells (Figure 4B) and fractions F6 and F13 were toxic to HaCaT cells (Figure 4C). Summary of all *in vitro* enzymatic assays and cell viability assays is presented in Supplementary Table 4. From these evaluations, we proceeded with compound isolation from fractions F7, F9, F11, and F12.

### 3.6 Structural elucidation

**Figure 6:**
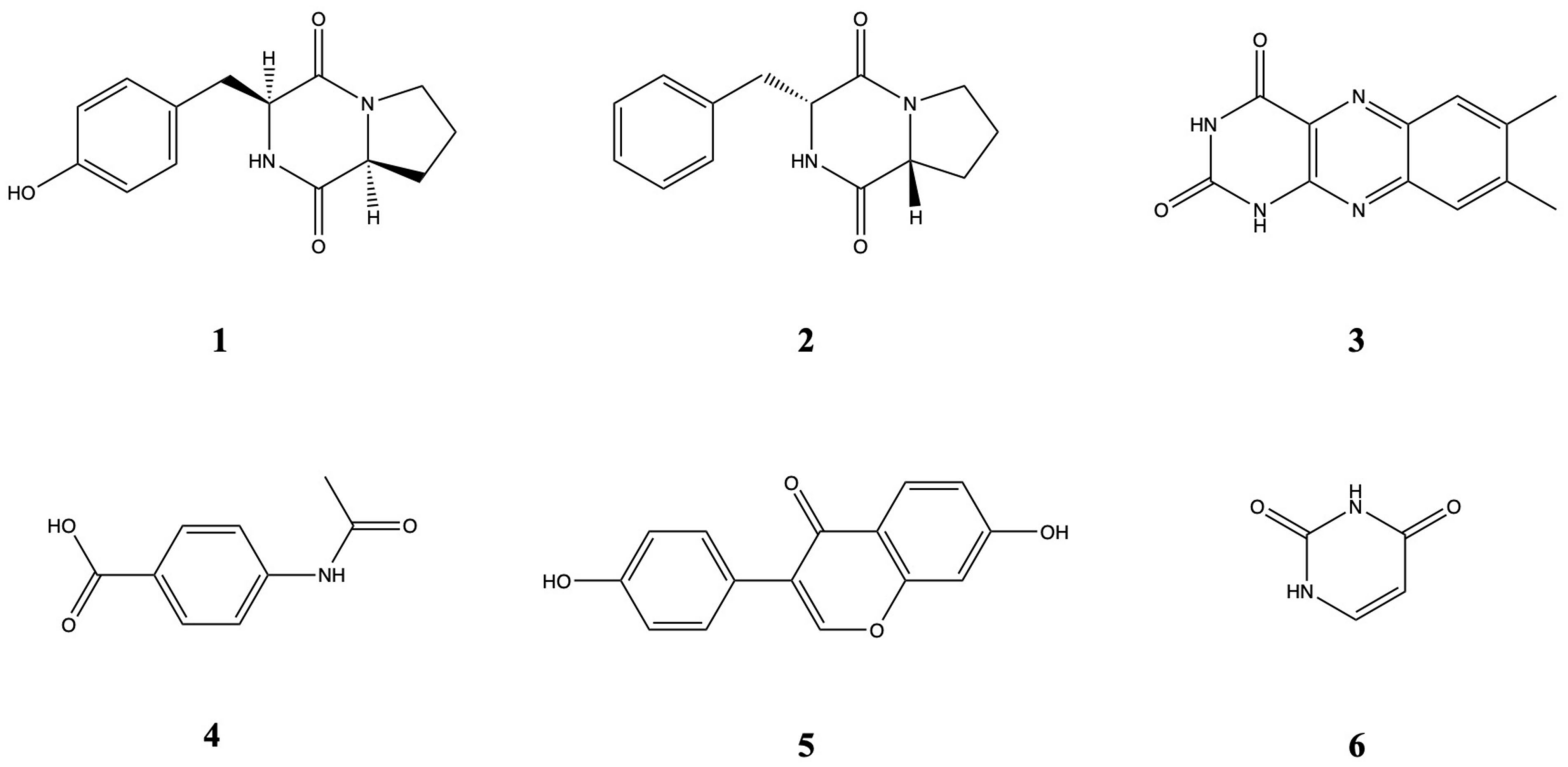
Chemical structures of compounds 1–6.

Isolations were carried out on the most bioactive and non-toxic fractions (F7, F9, F11, F12). The purification studies isolated Cyclo (L-proline-L-tyrosine), Cyclo (Pro-Phe) (2), Lumichrome (3), P-(acetylamino) benzoic acid (4), Daidzein (5), and Uracil (6). Chemical structures are presented in Figure 6. Their ^1^H NMR data are presented in Supplementary Figures 1–6.

Cyclo (L-proline-L-tyrosine) (1) was isolated as white amorphous powder. Its molecular formula, C_14_H_16_N_2_O_3_, was established by HRESIMS at *m/z* 261.1231 [M + H]^+^ (calcd for 261.1234). ^1^H NMR (600 MHz, CDCl_3_, *δ*, ppm, J/Hz): 1.89 (1H, m, H-12a), 1.91 (1H, m, H-7b), 2.01 (1H, m, H-12b), 2.34 (1H, m, H-7a), 2.75 (1H, dd, J = 14.5, 10.2, H-14b), 3.49 (1H, dd, J = 14.6, 3.6, H-14a), 3.57 (1H, m, H-13b), 3.64 (1H, m, H-13a), 4.08 (1H, t, J = 7.7, H-11), 4.22 (1H, dd, J = 8.8, 2.2, H-8), 5.99 (1H, s, NH-4, D_2_O exchangeable), 6.79 (2H, d, J = 8.2, H-3, 5), 7.07 (2H, d, J = 8.1, H-2, 6).

Cyclo (Pro-Phe) (2) was isolated as white amorphous powder. Its molecular formula, C_14_H_16_N_2_O_2_, was established by HRESIMS at *m/z* 245.1281 [M + H]^+^ (calcd for 245.1285).^1^H NMR (600 MHz, CDCl_3_, δ, ppm, J/Hz) δ 7.37 (t, *J* = 7.4 Hz, 2H), 7.30 (d, *J* = 6.8 Hz, 2H), 7.23 (d, J = 7.1, 2H), 5.61 (s, 1H), 4.27 (dd, 11.1, 2.5, 1H), 4.09 (t, *J* = 7.6, 1H), 3.70–3.53 (m, 3H), 2.78 (dd, *J* = 14.5, 10.7 Hz, 1H), 2.42–2.26 (m, 1H), 2.03 (m, 2H), 1.95–1.87 (m, 1H).

Lumichrome (3) was isolated as yellow powder. Its molecular formula, C_12_H_10_N_4_O_2_, established by HRESIMS at *m/z* 243,0822 [M + H]^+^ (calcd for 243.0877), ^1^H NMR (600 MHz, MeOD-*d*_4_, δ, ppm): 2.52 (3H, s, H-12), 2.54 (3H, s, H-11), 7.75 (1H, s, H-9), 7.94 (1H, s, H-6).

P-(acetylamino) benzoic acid (4) was isolated as white amorphous powder. Its molecular formula, C_9_H_9_NO_3_, established by HRESIMS at *m/z* 180.06557 [M + H]^+^, (calcd for 180.0655), ^1^H-NMR (600 MHz, MeOD-*d*4, δ, ppm, J/Hz): 2.15 (s, 3H), 7.65 (2H, d, J = 8.7), 7.95 (2H, d, *J* = 8.8).

Daidzein (5) was isolated as yellow powder. Its molecular formula, C_15_H_10_O_4_, established by HRESIMS at *m/z* 255.06491 [M + H]^+^ (calcd for 255.0652), ^1^H-NMR (600 MHz, MeOD-*d*4, δ, ppm, J/Hz): 6.84 (2H, d, *J* = 6.3, H-3′, 5′), 6.85 (1H, br.s, H-8), 6.94 (1H, dd, *J* = 8.8, 2.2, H-6), 7.37 (2H, d, J = 8.7, H-2′, 6′), 8.05 (1H, d, *J* = 8.8, H-5), 8.13 (1H, s, H-2).

Uracil (6) was isolated as white crystallized powder. Its molecular formula, C_4_H_4_N_2_O_2_, established by HRESIMS at *m/z* 113.0349 [M + H]^+^ (calcd for 113.0346), ^1^H-NMR (600 MHz, MeOD*-d*4, δ, ppm, J/Hz): 7.39 (1H, d, *J* = 7.7, H-6), 5.61 (1H, d, *J* = 7.4, H-5).

### 3.7 Bioactivity of pure molecules in cell models

**Figure 7:**
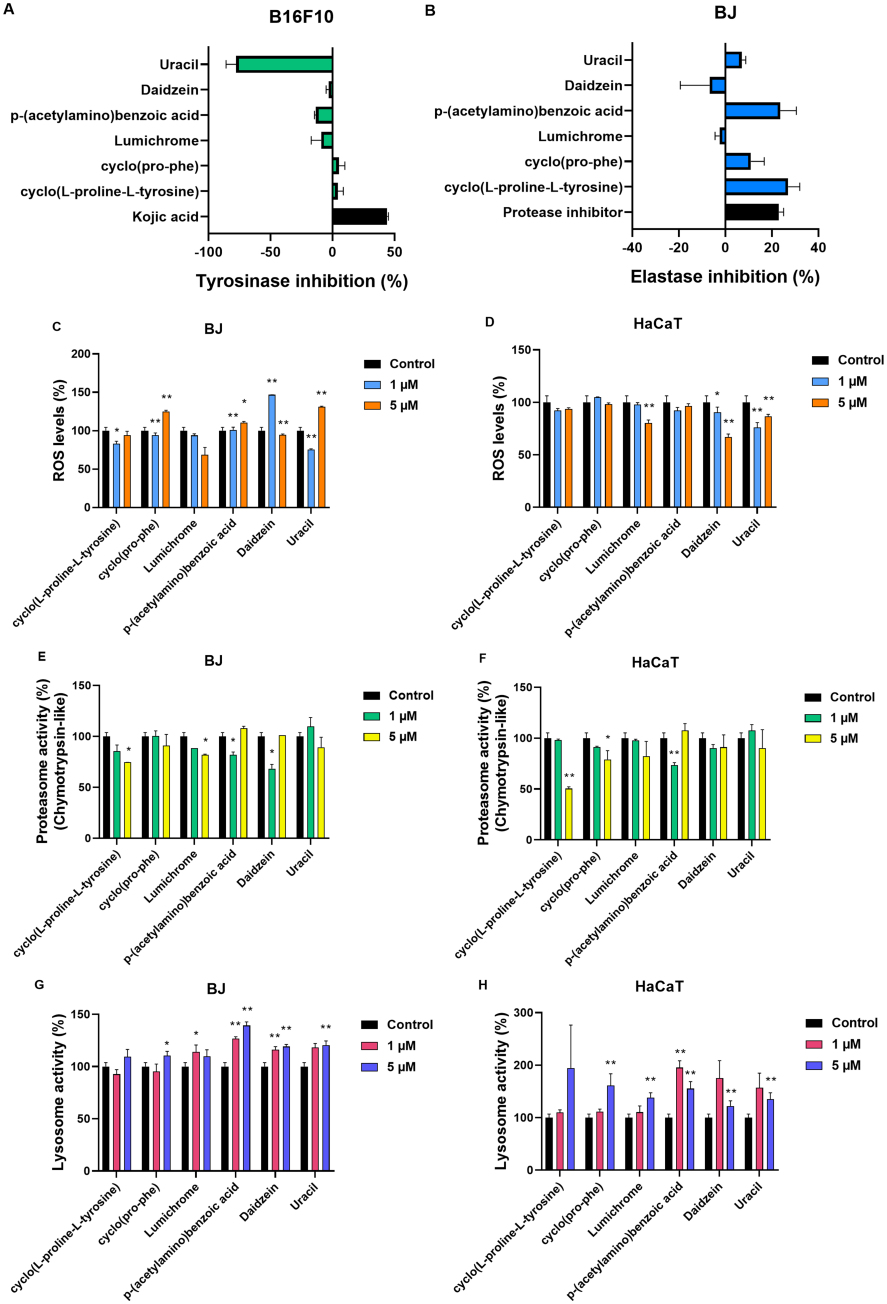
Compound testing in cell models. **(A)** Tyrosinase inhibition (%) for all compounds (2 μM) in B16-F10 melanocytes. Kojic acid (100 μg/mL) was used as positive control, ±SD. **(B)** Elastase inhibition (%) for all compounds (2 μM) in BJ fibroblasts. Protease inhibitor (1X) was used as positive control, ±SD. ROS (%) levels of **(C)** BJ and **(D)** HaCaT cells post 24 h treatment with the compounds at 1 μM and 5 μM. Proteasome activity levels (%, β5 subunit) of **(E)** BJ and **(F)** HaCaT cells. Both cell lines were treated for 24 h with the compounds at 1 μM and 5 μM. Lysosome activity levels (%) of **(G)** BJ and **(H)** HaCaT cells after 24 h treatment with the compounds at 1 μM and 5 μM. Control samples treated with DMSO and set to 100%. *p < 0.05, **p < 0.01, +SD.

These six metabolites [cyclo(L-proline-L-tyrosine) (1), cyclo(Pro-Phe) (2), Lumichrome (3), p-(acetylamino)benzoic acid (4), Daidzein (5), Uracil (6)] isolated from the most bioactive and non-toxic fractions (F7, F9, F11, F12) of the extract ATHUBA 292 were initially tested in cells for their potential antiaging, whitening and cytoprotective effects (Figure 7). Their potential activity against tyrosinase was thus tested in B16-F10 melanocytes, while their potential activity against elastase was tested in BJ cells. Finally, the compounds were studied for their effects against oxidative stress and the activation of key cellular anti-aging mechanisms, such as the ubiquitin-proteasome system and the autophagy-lysosome system, in BJ and HaCaT cells.

Testing of pure molecules for their antiaging and whitening activity determined that none of the six metabolites under study inhibited tyrosinase at a high rate in cellular assays (Figure 7A). In contrast, two of the pure molecules, [cyclo(L-proline-L-tyrosine)] (1) and [p-(acetylamino)benzoic acid] (4) showed an elastase inhibition rate of 26% and 23%, respectively, at a concentration of 2 μΜ (Figure 7B).

In addition to the anti-aging and whitening activity of metabolites, their potential antioxidant activity was tested on both BJ fibroblasts and HaCaT keratinocytes, as protection against oxidative stress is essential for cell survival. In BJ cells (Figure 7C), a decrease in ROS levels was observed after 24 h treatment with 1 μΜ and 5 μΜ of Lumichrome (3). A decrease in ROS levels was detected at the lowest 1 μΜ concentration of cyclo(Pro-Phe) (2), and Uracil (6), while 1μΜ of Daidzein (5) significantly increased ROS levels. At 5 μΜ concentration, ROS levels statistically increased compared to control cells, after treatment with cyclo(Pro-Phe) (2), p-(acetylamino)benzoic acid (4), and Uracil (6) respectively. In HaCaT keratinocytes (Figure 7C), the molecules Lumichrome (3), Daidzein (5) and Uracil (6) produced a statistically significant reduction in ROS levels compared to the control, while the rest did not seem to have a significant effect on the oxidative load of these cells (Figure 7D).

We also investigated how these metabolites influence the activity of major proteostatic processes, such as the activity β5 subunit within the 26S proteasome (Figures 7E,F) and cathepsin activity within the lysosome (Figures 7G,H). None of the molecules caused a statistically significant increase in the β5 proteasomic subunit levels in either of the cell lines. Instead, some molecules caused a decrease in its activity. More specifically, cyclo(L-proline-L-tyrosine) (1), Lumichrome (3), p-(acetylamino)benzoic acid (4) and Daidzein (5) reduced the activity of β5 proteasomic activity by up to 40% in BJ cells (Figure 7E), while in HaCaT cells (Figure 7F) a reduction of up to 50% was observed in the β5 proteasome subunit activity after treatment with cyclo(L-proline-L-tyrosine) (1), cyclo(Pro-Phe) (2) and p-(acetylamino)benzoic acid (4) for 24 h. Finally, the effect of metabolites on the autophagy-lysosome pathway and specifically on the activity of lysosomal B and L cathepsins, was studied in the two cell lines (Figures 7G,H). In BJ cells an increase at the studied levels was caused by all compounds except cyclo(L-proline-L-tyrosine) (1) (Figure 7G). In HaCaT cells all metabolites increased lysosomal activity (Figure 7H).

### 3.8 *In vivo* bioactivity effects of pure molecules on *Drosophila melanogaster*

The most bioactive metabolites, according to the assays conducted in cell models, were used as food supplements in *Drosophila melanogaster,* to assess possible antiaging properties at an organismal level. More specifically, according to the availability of each metabolite, the pure molecules that were studied were cyclo(L-proline-L-tyrosine) (1), cyclo(Pro-Phe) (2), p-(acetylamino) benzoic acid (4) and Uracil (6) at concentrations of 1 μΜ and 10 μΜ.

**Figure 8:**
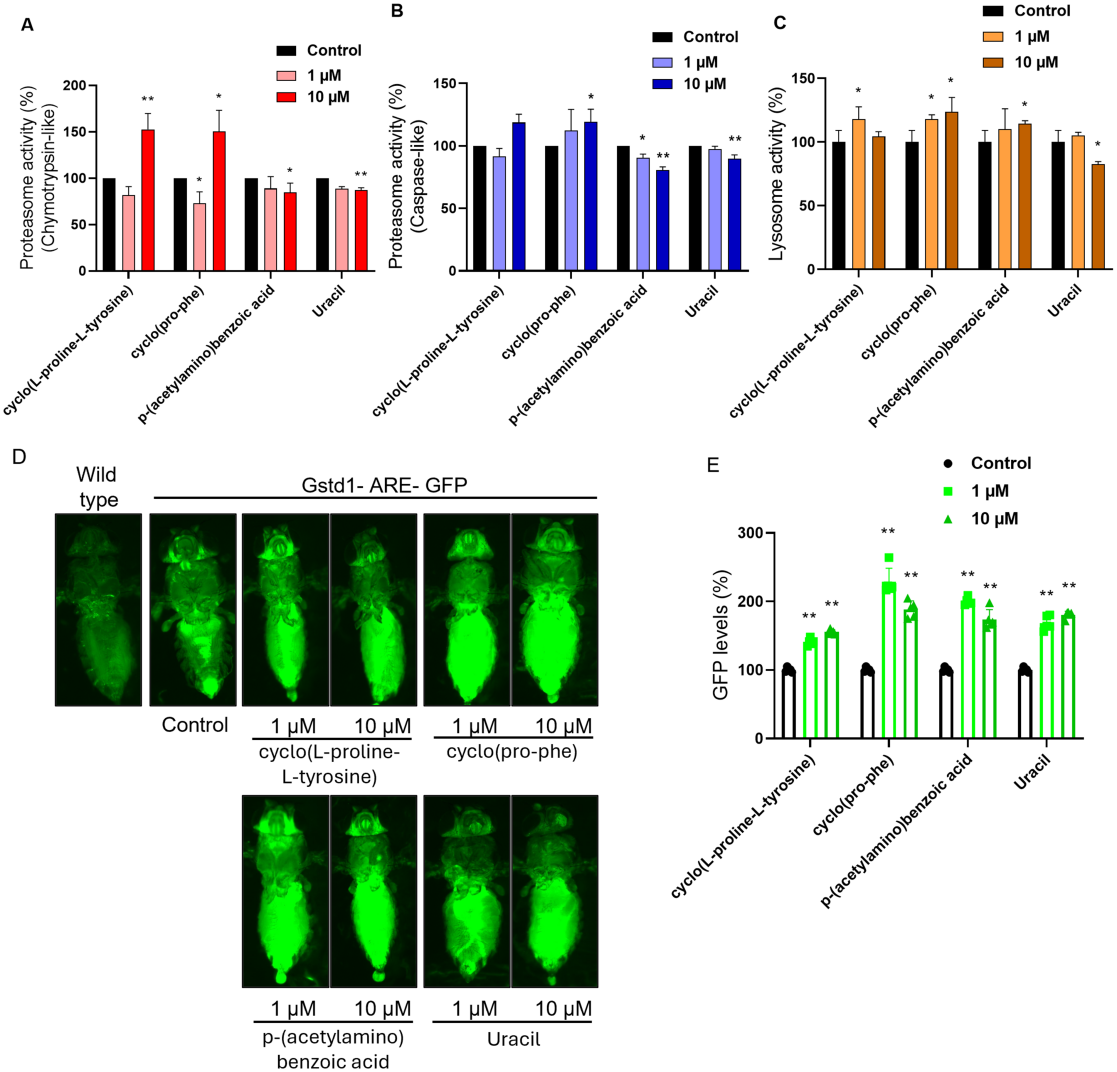
Measurement of compound ability to activate major response pathways in an *in vivo* model. Proteasome activity levels (%) measured by the activity of **(A)** the β5 subunit (chymotrypsin-like activity) and **(B)** the β1 subunit (caspase-like activity) of the complex, in somatic tissues of young flies (7–10 days old). **(C)** Lysosome cathepsin levels (%) in somatic tissues of young flies (7–10 days old). **(D)** Imaging of GFP levels in GstD1-ARE: GFP transgenic flies in comparison with wild type flies and untreated control. **(E)** Measurement of GFP levels (%, RFUs/μg protein) of somatic tissues of young (7–10 days) transgenic GstD1-ARE: GFP flies. Non-treated GstD1-ARE: GFP values set to 100%. Compound concertation: 1 μM and 10 μM. Untreated control population supplemented with DMSO. *p < 0.05, **p < 0.01, +SD.

In these experiments the levels of proteasome activity were assessed and more specifically the activity of β5 and β1 subunits that have chymotrypsin and caspase activity, respectively. There was a statistically significant increase in the β5 proteasome activity in the samples where 10 μΜ of cyclo(L-proline-L-tyrosine) (1) or cyclo(Pro-Phe) (2) were administrated, while conversely when p-(acetylamino)benzoic acid (4) or Uracil (6) was supplemented in the food, there was a statistically significant decrease (Figure 8A). The molecules p-(acetylamino)benzoic acid (4) and Uracil (6) also lead to a decrease in the activity of β1 proteasomic subunit, when supplemented in the food, while cyclo(pro-phe) (2) increased its activity at 10 μΜ concentration (Figure 8B).

Following that, we studied the activity of cathepsins, proteases that mostly reside in the lysosome, in somatic tissues of wild type flies that were cultured with or without the administration of 1 μΜ or 10 μΜ of each metabolite chosen from the cell-based assays. The results indicated that the cyclo(L-proline-L-tyrosine) (1), cyclo(Pro-Phe) (2), and p-(acetylamino) benzoic acid (4) can increase the activity of the lysosomal cathepsins, while Uracil (6) reduced it to 82% (Figure 8C).

Finally, all the metabolites can induce CncC, *D.melanogaster’s* ortholog for the Nrf2 transcription factor, and thereby trigger antioxidant responses in the organism (Figure 8D). The activation level of CncC varies by metabolite type (Figure 8D) and is dose dependent (Figure 8E), with cyclo(Pro-Phe) (2) causing the highest increase in GFP levels.

## 4 Discussion

Aging is characterized by an imbalance in proteome functionality, which results from an impairment in the coordinated production and maintenance of correctly folded polypeptides, or the degradation of misfolded proteins (López-Otín et al., 2023). The loss of proteostasis in the cell and the accumulation of senescent cells can eventually lead to physiological changes such as wrinkles, dark spots, or more severe conditions, such as cancer or neurodegenerative diseases (López-Otín et al., 2023; Gu et al., 2020). There have been extensive efforts over the years to find novel compounds which will not only lead to aesthetic improvements but will also help extend a healthy lifespan. There is a considerable interest in preventing or delaying skin aging and photoaging in particular; in recent years the cosmetic industry has been expanding at a rate of around 15%, and it is predicted to continue to grow (Bilal and Iqbal, 2020).

This work describes the screening of a large number of actinobacterial isolates from Greek ecosystems for their ability to generate potent extracts with promising anti-tyrosinase or anti-elastase activity. Among the studied extracts, −tyrosine inhibition was over 21 times more common compared to elastase inhibition. This study also determined that ethyl acetate is superior to methanol for the isolation of bioactive metabolites, as significantly more ethyl acetate extracts demonstrated anti-tyrosinase and anti-elastase activity. In addition, methanol was more indiscriminate in the molecules it extracted, which makes identifying specific bioactive molecules more difficult.

The absence of anti-tyrosinase activity observed in the non-cytotoxic fractions following bioactivity-guided fractionation, despite the strong inhibition detected in the crude extract, may be attributed to several factors. One possible explanation is that the observed activity in the extract arises from synergistic interactions among multiple constituents, which are disrupted upon chromatographic separation. Such synergism is well-recognized in microbial extracts, where individual compounds may display limited activity when tested in isolation. Additionally, the active component(s) may be present in low abundance, spread across multiple fractions, or co-eluting in cytotoxic compounds that were not pursued in subsequent analyses due to their effect on cell viability. It is also possible that matrix effects in crude extract enhanced the solubility, chemical stability, or bioavailability of metabolites, facilitating stronger inhibition in the unfractionated material. These considerations highlight the complexity of bioactivity-guided isolation and underscore the need for complementary strategies such as fraction recombination, untargeted metabolomics and microfractionation-bioassay correlation to better preserve and interpret bioactivity throughout the purification process.

Testing the activity of the extracts led to the isolation and structure elucidation of six secondary metabolites: Cyclo(L-Pro-L-Tyr) (1), also known as maculosin-1, is a diketipiperazine with a strong inhibitory effect on the enzyme tyrosinase. Molecular docking simulation studies have demonstrated that this compound exhibits promising results in studies with tyrosinase originating from mushrooms, offering support for the idea that it is a low toxicity tyrosinase inhibitor (Sekino et al., 2024). Maculosin has shown promising antioxidant and anticancer activity, though there are not enough data regarding its effects on human elastase (Paudel et al., 2021; Jinendiran et al., 2020). Cyclo(Pro-Phe) (2) has been primarily examined for its quorum sensing and signaling roles in bacteria, and its anti-tyrosinase activity has not been thoroughly evaluated. While cyclo(Pro-Phe) has demonstrated promising applications in these areas, specific studies on its anti-tyrosinase activity are limited and further research would be needed to confirm any significant impact on tyrosinase activity (Son et al., 2024). Similarly, there are limited data on its effects on elastase activity, even though our results suggest a possible inhibitory activity. Interestingly, there are indications in publications that cyclo(Pro-Phe) shows anticancer activity against human cancer cell lines (Chen et al., 2018). Lumichrome (3) is a derivative of riboflavin and there is no reporting of anti-tyrosinase or anti-elastase activity in the current scientific literature. This compound has shown activity in biological systems, particularly in quorum sensing and other microbial processes but its specific inhibition against those enzymes does not appear to have been investigated (Baber et al., 2023). In our study, lumichrome, did not show any inhibitory action against elastase or tyrosinase, but it caused a decrease of reactive oxygen species and an increase of cathepsins’ activity in HaCaT keratinocytes. P-(acetylamino) benzoic acid (4) has not been reported to have specific anti-tyrosinase activity in the scientific literature. There are no existing studies regarding its effects on elastase, however we identified a high level of elastase inhibition. Benzoic acid derivatives have been studied for their potential inhibitory effects on tyrosinase. While p-(acetylamino) benzoic acid has not been noted as a significant tyrosinase inhibitor, related benzoic acid derivatives have shown some promising results in enzyme inhibition, particularly when tested in food preservation or cosmetic applications for skin whitening. These effects often rely on the specific chemical modifications of the benzoic acid backbone, which influence its binding affinity to the enzyme (Baber et al., 2023; Obaid et al., 2021). Daidzein (5) is an isoflavone commonly found in soybeans which has shown moderate anti-tyrosinase activity. Studies have reported that daidzein exhibits an IC_50_ value of around 203 μM, which indicates its inhibitory potential against tyrosinase, although it is not as potent as other isoflavones such as genistein or certain derivatives like 7,8,4′-trihydroxyisoflavone. These derivatives can be more effective, with much lower IC_50_ values, showing stronger tyrosinase inhibition (Fernandes and Kerkar, 2017; Obaid et al., 2021). Moreover, while this compound did not show any inhibition of elastase in human cells, it exhibits suppressive activity against articular elastase in rats, as well as anti-inflammatory activity (Ahmad et al., 2016). Daidzein has been reported in the literature to have multiple roles in aging and disease. It has shown biological activity in multiple pathologies, as well as antioxidant and anti-inflammatory effects (Ahmad et al., 2024). Uracil (6) does not have any published reports on its effects on tyrosinase or elastase activity in humans. In this study, uracil inhibits elastase in human cells. There is extensive research regarding this compound and its biological roles, while there are, in comparison, a few studies where this compound is exogenously administrated. There are also numerous studies about the biological activity of its derivatives, like 5-fluo-uracil and their pharmacological roles (Pałasz and Cież, 2015). Finally, this compound was also researched for its involvement in quorum sensing (Ueda et al., 2009).

Several actinomycete-derived metabolites have been reported with activities comparable to those observed in our study. Osmanicin, a polyketide alkaloid isolated from *Streptomyces osmaniensis*, displayed significant elastase inhibition in human dermal fibroblasts (IC_50_ = 3.7 μM), highlighting the capacity of microbial metabolites to modulate proteolytic enzymes associated with skin aging (Samy et al., 2019). Αdditionally previous screening of our research group on 614 microbial actinomycete extracts for the discovery of tyrosinase inhibitors, the EtOAc extract of a *Streptomyces* sp. CA-129531 strain, exhibited in cell free and cell-based assays strong biological activity (IC_50_ value of 63 μg/mL). Scaled-up production in a bioreactor led to the isolation among others of six trichostatine derivatives and four diketopiperazines (Georgousaki et al., 2020). Although trichostatin A, (IC_50_ 2.18 μΜ) showed six times stronger anti-tyrosinase activity than the positve control kojic acid (IC_50_ 14.07 μΜ), the isolated diketopiperazines were not evaluated due to their variability in cytotoxicity. In contrast, the compounds isolated in our study (e.g., Cyclo(L-Pro-L-Tyr) and p-(acetylamino)benzoic acid) exhibited moderate elastase inhibition coupled with low cytotoxicity and importantly, did not retain significant tyrosinase inhibition in their purified forms. These features highlight the favorable bioactivity of the isolated compounds, which demonstrate moderate yet selective enzyme inhibition, low cytotoxicity and minimal off-target effects.

Administration of selected isolated molecules as food supplements in *D. melanogaster*’s food triggered the transcription of GstD1-GFP reporter downstream of AREs responsive to CncC, the invertebrate’s ortholog to Nuclear factor erythroid 2-related factor 2 (Nrf2). Nrf2 is a transcription factor responsible, among others, for redox homeostasis, and it is found to be downregulated during aging (Tsakiri et al., 2019). Since sun exposure and pollution generate reactive oxygen species that degrade collagen and impair its production, which accelerates skin aging (Barnawi and Barnawi, 2021), compounds with antioxidant activity can potentially be used to delay skin aging or as food supplements to reduce oxidative stress. This study also determined that two compounds, cyclo(L-Pro-L-Tyr) (1) and cyclo(Pro-Phe) (2), enhanced both proteasome and cathepsins activity on an organismal level. This suggests that the administration of those compounds may help increase autophagy and the activity of the ubiquitin-proteasome system, processes that contribute to misfolded protein turnover. The activity of both those components of the proteostasis network declines as organisms age, while their induction has been linked to healthspan and lifespan increase (Taylor and Dillin, 2011). Since it has been suggested that autophagy is capable of delaying the photoaging of skin caused by solar ultraviolet radiation (Wang et al., 2019), and the ubiquitin-proteasome system degrades tyrosinase (Ando et al., 2009), the activation of the cellular mechanisms mediating these processes could, respectively, help delay skin aging and whiten the skin.

In conclusion, our findings indicate that Greek microbial diversity and its derived extracts are a promising source for small molecules that can counteract certain effects of skin aging via tyrosinase inhibition, elastase inhibition, antioxidant activity and activation of autophagy and the ubiquitin-proteasome system. Therefore, these actinobacterial extracts and isolated compounds have potential use in delaying the effects of skin aging and further research is needed for the integration of these molecules into cosmeceutical products or food supplements. The potential effectiveness of these compounds is supported by previous research in this area, as a multifunctional cosmetic cream with anti-tyrosinase and anti-aging activities has been previously generated using bioactive materials from streptomycetes (Dahal et al., 2020; Dahal et al., 2017). Further investigation might lead to their use in drugs or formulations for the treatment of various aging-related skin conditions like wrinkles, photo-aging, skin dryness, dark spots, hair damage and hyperpigmentation. In addition further study of these compounds for their UV absorption capacity or photoprotective properties could be performed since these are related to skin aging.

## Data Availability

The datasets presented in this study are publicly available. This data can be found at: https://www.ncbi.nlm.nih.gov/genbank, accession numbers PV793531-PV793534.

## Footnotes

^1^ https://jgi.doe.gov/wp-content/uploads/2014/02/JGI-Bacterial-DNA-isolation-CTAB-Protocol-2012.pdf

^2^ http://blast.ncbi.nlm.nih.gov/Blast.cgi
