# Supplementary material for: Exploring Greek actinobacterial biodiversity for the discovery of bioactive metabolites with skin antiaging potential
Source: Front Microbiol. 2025 Oct 9;16:1649460. doi: 10.3389/fmicb.2025.1649460 (PMC12546214; doi:10.3389/fmicb.2025.1649460)
Supplement: Supplementary file 1 [file Table_1.DOCX]

**Exploring Actinobacteria of Greek Biodiversity for the Discovery of Bioactive Metabolites with Skin Antiaging Potential**

**Konstantinos Gaitanis†^1^, Eirini Gkogkou†^2^, Paris Laskaris†^3^, Nikolaοs Tsafantakis^1^, Despoina D. Gianniou^2^, Stavroula I. Kaili^3^, Georgia C. Ntroumpogianni^3^, Aikaterini Theodosopoulou^2^, Nikola Milic^1^, Dimitris G. Hatzinikolaou^3^, Nikolas Fokialakis*^1^, Ioannis P. Trougakos*^2^, Amalia D. Karagouni*^3^,**

^1^ Division of Pharmacognosy & Chemistry of Natural Products, Department of Pharmacy, National and Kapodistrian University of Athens, Zografou Campus, 15771 Athens, Greece

^2^ Division of Cell Biology and Biophysics, Department of Biology, National and Kapodistrian University of Athens, Zografou Campus, 15784 Athens, Greece

^3^ Division of Botany, Department of Biology, National and Kapodistrian University of Athens, Zografou Campus, 15784 Athens, Greece

†These authors have contributed equally to this work.

*** Correspondence:**Corresponding Authors
[akar@biol.uoa.gr](mailto:akar@biol.uoa.gr), [fokialakis@pharm.uoa.gr](mailto:fokialakis@pharm.uoa.gr), [itrougakos@biol.uoa.gr](mailto:itrougakos@biol.uoa.gr)

**Supplementary Table 1**: Isolation locations and sources of strains used in this study.

| Collection area | Sample from | Strain |
| --- | --- | --- |
| Eleusis | Soil | ATHUBA 2503 |
|  |  | ATHUBA 2492 |
|  |  | ATHUBA 2497 |
|  |  | ATHUBA 2516 |
|  |  | ATHUBA 2517 |
|  |  | ATHUBA 2507 |
| Oeta | Rhizosphere *Veronica oetaea* | ATHUBA 490 |
|  |  | ATHUBA 491 |
|  |  | ATHUBA 492 |
|  |  | ATHUBA 493 |
|  |  | ATHUBA 494 |
| Parnitha | Rhizosphere *Abies cephalonica* | ATHUBA 603 |
|  |  | ATHUBA 781 |
|  |  | ATHUBA 604 |
|  |  | ATHUBA 782 |
|  |  | ATHUBA 605 |
|  |  | ATHUBA 606 |
|  |  | ATHUBA 601 |
|  |  | ATHUBA 602 |
| Samos | Rhizosphere evergreen shrub | ATHUBA 210 |
|  |  | ATHUBA 212 |
|  |  | ATHUBA 214 |
|  |  | ATHUBA 215 |
|  |  | ATHUBA 216 |
|  |  | ATHUBA 217 |
|  |  | ATHUBA 218 |
|  |  | ATHUBA 219 |
|  |  | ATHUBA 234 |
|  |  | ATHUBA 221 |
|  |  | ATHUBA 222 |
|  |  | ATHUBA 223 |
|  |  | ATHUBA 224 |
|  |  | ATHUBA 225 |
|  |  | ATHUBA 213 |
|  |  | ATHUBA 226 |
|  |  | ATHUBA 236 |
|  |  | ATHUBA 227 |
|  |  | ATHUBA 228 |
|  |  | ATHUBA 245 |
|  |  | ATHUBA 211 |
|  |  | ATHUBA 229 |
|  |  | ATHUBA 230 |
|  |  | ATHUBA 231 |
|  |  | ATHUBA 232 |
|  |  | ATHUBA 233 |
|  |  | ATHUBA 764 |
|  |  | ATHUBA 590 |
|  |  | ATHUBA 238 |
|  |  | ATHUBA 239 |
|  |  | ATHUBA 240 |
|  |  | ATHUBA 241 |
|  |  | ATHUBA 242 |
|  |  | ATHUBA 243 |
|  |  | ATHUBA 244 |
|  |  | ATHUBA 767 |
|  |  | ATHUBA 220 |
| Kaisariani | Rhizosphere woody shrub | ATHUBA 608 |
|  |  | ATHUBA 609 |
|  |  | ATHUBA 610 |
|  |  | ATHUBA 611 |
|  |  | ATHUBA 612 |
|  |  | ATHUBA 613 |
|  |  | ATHUBA 614 |
|  |  | ATHUBA 615 |
|  |  | ATHUBA 616 |
|  |  | ATHUBA 607 |
|  |  | ATHUBA 783 |
|  |  | ATHUBA 624 |
|  |  | ATHUBA 625 |
|  |  | ATHUBA 626 |
|  |  | ATHUBA 628 |
|  |  | ATHUBA 629 |
|  |  | ATHUBA 630 |
|  |  | ATHUBA 632 |
|  |  | ATHUBA 633 |
|  |  | ATHUBA 784 |
|  |  | ATHUBA 622 |
|  |  | ATHUBA 623 |
|  |  | ATHUBA 637 |
|  |  | ATHUBA 638 |
|  |  | ATHUBA 640 |
|  |  | ATHUBA 641 |
| Schinias | Rhizosphere coastal plants | ATHUBA 800 |
|  |  | ATHUBA 810 |
|  |  | ATHUBA 811 |
|  |  | ATHUBA 812 |
|  |  | ATHUBA 813 |
|  |  | ATHUBA 814 |
|  |  | ATHUBA 815 |
|  |  | ATHUBA 816 |
|  |  | ATHUBA 817 |
|  |  | ATHUBA 818 |
|  |  | ATHUBA 819 |
|  |  | ATHUBA 820 |
|  |  | ATHUBA 804 |
|  |  | ATHUBA 805 |
|  |  | ATHUBA 806 |
|  |  | ATHUBA 807 |
|  |  | ATHUBA 808 |
|  |  | ATHUBA 809 |
| Crete | Rhizosphere *Ceratonia siliqua* | ATHUBA 178 |
|  |  | ATHUBA 143 |
|  |  | ATHUBA 151 |
|  |  | ATHUBA 756 |
|  |  | ATHUBA 757 |
|  |  | ATHUBA 758 |
|  |  | ATHUBA 153 |
|  |  | ATHUBA 154 |
|  |  | ATHUBA 155 |
|  |  | ATHUBA 156 |
|  |  | ATHUBA 157 |
|  |  | ATHUBA 144 |
|  |  | ATHUBA 158 |
|  |  | ATHUBA 159 |
|  |  | ATHUBA 759 |
|  |  | ATHUBA 160 |
|  |  | ATHUBA 161 |
|  |  | ATHUBA 163 |
|  |  | ATHUBA 164 |
|  |  | ATHUBA 760 |
|  |  | ATHUBA 755 |
|  |  | ATHUBA 165 |
|  |  | ATHUBA 166 |
|  |  | ATHUBA 167 |
|  |  | ATHUBA 169 |
|  |  | ATHUBA 170 |
|  |  | ATHUBA 172 |
|  |  | ATHUBA 173 |
|  |  | ATHUBA 145 |
|  |  | ATHUBA 174 |
|  |  | ATHUBA 175 |
|  |  | ATHUBA 176 |
|  |  | ATHUBA 761 |
|  |  | ATHUBA 177 |
|  |  | ATHUBA 179 |
|  |  | ATHUBA 180 |
|  |  | ATHUBA 181 |
|  |  | ATHUBA 182 |
|  |  | ATHUBA 146 |
|  |  | ATHUBA 183 |
|  |  | ATHUBA 185 |
|  |  | ATHUBA 186 |
|  |  | ATHUBA 188 |
|  |  | ATHUBA 147 |
|  |  | ATHUBA 148 |
|  |  | ATHUBA 149 |
|  |  | ATHUBA 150 |
| Hymettus | Rhizosphere *Cistus creticus* | ATHUBA 2540 |
|  |  | ATHUBA 2542 |
|  |  | ATHUBA 2543 |
|  |  | ATHUBA 2544 |
|  |  | ATHUBA 2545 |
|  |  | ATHUBA 2546 |
|  |  | ATHUBA 2547 |
|  |  | ATHUBA 2549 |
|  |  | ATHUBA 2532 |
|  |  | ATHUBA 2550 |
|  |  | ATHUBA 2551 |
|  |  | ATHUBA 2552 |
|  |  | ATHUBA 2553 |
|  |  | ATHUBA 2555 |
|  |  | ATHUBA 2558 |
|  |  | ATHUBA 2559 |
|  |  | ATHUBA 2533 |
|  |  | ATHUBA 2560 |
|  |  | ATHUBA 2561 |
|  |  | ATHUBA 2562 |
|  |  | ATHUBA 2563 |
|  |  | ATHUBA 2564 |
|  |  | ATHUBA 2535 |
|  |  | ATHUBA 2538 |
| Crete | Rhizosphere coniferous trees | ATHUBA 455 |
|  |  | ATHUBA 367 |
|  |  | ATHUBA 368 |
|  |  | ATHUBA 369 |
|  |  | ATHUBA 371 |
|  |  | ATHUBA 375 |
|  |  | ATHUBA 376 |
|  |  | ATHUBA 377 |
|  |  | ATHUBA 378 |
|  |  | ATHUBA 379 |
|  |  | ATHUBA 381 |
|  |  | ATHUBA 361 |
|  |  | ATHUBA 387 |
|  |  | ATHUBA 388 |
|  |  | ATHUBA 390 |
|  |  | ATHUBA 391 |
|  |  | ATHUBA 392 |
|  |  | ATHUBA 362 |
|  |  | ATHUBA 395 |
|  |  | ATHUBA 396 |
|  |  | ATHUBA 411 |
|  |  | ATHUBA 771 |
|  |  | ATHUBA 413 |
|  |  | ATHUBA 364 |
|  |  | ATHUBA 421 |
|  |  | ATHUBA 424 |
|  |  | ATHUBA 425 |
|  |  | ATHUBA 426 |
|  |  | ATHUBA 427 |
|  |  | ATHUBA 428 |
|  |  | ATHUBA 438 |
|  |  | ATHUBA 442 |
|  |  | ATHUBA 447 |
|  |  | ATHUBA 449 |
|  |  | ATHUBA 452 |
|  |  | ATHUBA 453 |
| Athens National Garden | Rhizosphere *Cupressus sempervirens* | ATHUBA 933 |
|  |  | ATHUBA 941 |
|  |  | ATHUBA 942 |
|  |  | ATHUBA 943 |
|  |  | ATHUBA 944 |
|  |  | ATHUBA945 |
|  |  | ATHUBA 946 |
|  |  | ATHUBA 947 |
|  |  | ATHUBA 948 |
|  |  | ATHUBA 934 |
|  |  | ATHUBA 935 |
|  |  | ATHUBA 936 |
|  |  | ATHUBA 938 |
|  |  | ATHUBA 940 |
| Athens National Garden | Rhizosphere *Fitolaca americana* | ATHUBA 885 |
|  |  | ATHUBA 894 |
|  |  | ATHUBA 895 |
|  |  | ATHUBA 896 |
|  |  | ATHUBA 898 |
|  |  | ATHUBA 899 |
|  |  | ATHUBA 900 |
|  |  | ATHUBA 901 |
|  |  | ATHUBA 902 |
|  |  | ATHUBA 903 |
|  |  | ATHUBA 886 |
|  |  | ATHUBA 904 |
|  |  | ATHUBA 887 |
|  |  | ATHUBA 889 |
|  |  | ATHUBA 893 |
| Crete | Rhizosphere *Pinus brutia* | ATHUBA 118 |
|  |  | ATHUBA 119 |
|  |  | ATHUBA 120 |
|  |  | ATHUBA 123 |
|  |  | ATHUBA 124 |
|  |  | ATHUBA 126 |
|  |  | ATHUBA 798 |
|  |  | ATHUBA 132 |
|  |  | ATHUBA 134 |
|  |  | ATHUBA 135 |
|  |  | ATHUBA 137 |
|  |  | ATHUBA 754 |
|  |  | ATHUBA 140 |
|  |  | ATHUBA 120 |
| Kastos | Soil | ATHUBA 646 |
|  |  | ATHUBA 646 |
|  |  | ATHUBA 648 |
|  |  | ATHUBA 649 |
|  |  | ATHUBA 650 |
|  |  | ATHUBA 652 |
|  |  | ATHUBA 651 |
|  |  | ATHUBA 651 |
|  |  | ATHUBA 642 |
|  |  | ATHUBA 653 |
|  |  | ATHUBA 788 |
|  |  | ATHUBA 643 |
|  |  | ATHUBA 654 |
|  |  | ATHUBA 655 |
|  |  | ATHUBA 789 |
|  |  | ATHUBA 656 |
|  |  | ATHUBA 790 |
|  |  | ATHUBA 646 |
|  |  | ATHUBA 792 |
|  |  | ATHUBA 793 |
|  |  | ATHUBA 645 |
| Kalavryta | Rhizosphere *Euphorbia myrsinites* | ATHUBA 2165 |
|  |  | ATHUBA 2174 |
|  |  | ATHUBA 2176 |
|  |  | ATHUBA 2177 |
|  |  | ATHUBA 2180 |
|  |  | ATHUBA 2181 |
|  |  | ATHUBA 2182 |
|  |  | ATHUBA 2166 |
|  |  | ATHUBA 2184 |
|  |  | ATHUBA 2185 |
|  |  | ATHUBA 2167 |
|  |  | ATHUBA 2168 |
|  |  | ATHUBA 2172 |
| Kalavryta | Rhizosphere *Conium* sp. | ATHUBA 2187 |
|  |  | ATHUBA 2196 |
|  |  | ATHUBA 2197 |
|  |  | ATHUBA 2198 |
|  |  | ATHUBA 2200 |
|  |  | ATHUBA 2201 |
|  |  | ATHUBA 2202 |
|  |  | ATHUBA 2203 |
|  |  | ATHUBA 2206 |
|  |  | ATHUBA 2208 |
|  |  | ATHUBA 2209 |
|  |  | ATHUBA 2187 |
|  |  | ATHUBA 2189 |
|  |  | ATHUBA 2190 |
|  |  | ATHUBA 2192 |
|  |  | ATHUBA 2193 |
|  |  | ATHUBA 2195 |
| Kalavryta | Rhizosphere Cyclamen graecum | ATHUBA 2211 |
|  |  | ATHUBA 2220 |
|  |  | ATHUBA 2221 |
|  |  | ATHUBA 2222 |
|  |  | ATHUBA 2223 |
|  |  | ATHUBA 2224 |
|  |  | ATHUBA 2225 |
|  |  | ATHUBA 2227 |
|  |  | ATHUBA 2228 |
|  |  | ATHUBA 2212 |
|  |  | ATHUBA 2214 |
|  |  | ATHUBA 2215 |
|  |  | ATHUBA 2216 |
|  |  | ATHUBA 2218 |
|  |  | ATHUBA 2219 |
| Kalavryta | Rhizosphere *Umbilicus rupestris* | ATHUBA 2238 |
|  |  | ATHUBA 2240 |
|  |  | ATHUBA 2241 |
|  |  | ATHUBA 2242 |
|  |  | ATHUBA 2244 |
|  |  | ATHUBA 2230 |
|  |  | ATHUBA 2249 |
|  |  | ATHUBA 2250 |
|  |  | ATHUBA 2251 |
|  |  | ATHUBA 2230 |
|  |  | ATHUBA 2231 |
|  |  | ATHUBA 2232 |
| Kastos | Soil | ATHUBA 694 |
|  |  | ATHUBA 697A |
|  |  | ATHUBA 698 |
|  |  | ATHUBA 699 |
|  |  | ATHUBA 700A |
|  |  | ATHUBA 701 |
|  |  | ATHUBA 702 |
|  |  | ATHUBA 704 |
|  |  | ATHUBA 745 |
|  |  | ATHUBA 746 |
|  |  | ATHUBA 709 |
|  |  | ATHUBA 711 |
|  |  | ATHUBA 691A |
| Crete | Rhizosphere *Punica granatum* | ATHUBA 1855 |
|  |  | ATHUBA 1864 |
|  |  | ATHUBA 1865 |
|  |  | ATHUBA 1866 |
|  |  | ATHUBA 1867 |
|  |  | ATHUBA 1868 |
|  |  | ATHUBA 1869 |
|  |  | ATHUBA 1872 |
|  |  | ATHUBA 1873 |
|  |  | ATHUBA 1856 |
|  |  | ATHUBA 1874 |
|  |  | ATHUBA 1875 |
|  |  | ATHUBA 1877 |
|  |  | ATHUBA 1879 |
|  |  | ATHUBA 1882 |
|  |  | ATHUBA 1883 |
|  |  | ATHUBA 1884 |
|  |  | ATHUBA 1886 |
|  |  | ATHUBA 1888 |
|  |  | ATHUBA 1890 |
|  |  | ATHUBA 1859 |
|  |  | ATHUBA 1861 |
|  |  | ATHUBA 1862 |
|  |  | ATHUBA 1863 |
| Crete | Rhizosphere *Cistus ladanifer* | ATHUBA 2133 |
|  |  | ATHUBA 2134 |
|  |  | ATHUBA 2135 |
|  |  | ATHUBA 2136 |
|  |  | ATHUBA 2137 |
|  |  | ATHUBA 2138 |
|  |  | ATHUBA 2139 |
|  |  | ATHUBA 2140 |
|  |  | ATHUBA 2141 |
|  |  | ATHUBA 2142 |
|  |  | ATHUBA 2125 |
|  |  | ATHUBA 2127 |
|  |  | ATHUBA 2128 |
|  |  | ATHUBA 2130 |
| Crete | Rhizosphere *Cercis siliquastrum* | ATHUBA 2143 |
|  |  | ATHUBA 2152 |
|  |  | ATHUBA 2154 |
|  |  | ATHUBA 2155 |
|  |  | ATHUBA 2157 |
|  |  | ATHUBA 2158 |
|  |  | ATHUBA 2159 |
|  |  | ATHUBA 2160 |
|  |  | ATHUBA 2161 |
|  |  | ATHUBA 2162 |
|  |  | ATHUBA 2157 |
|  |  | ATHUBA 2164 |
|  |  | ATHUBA 2165 |
|  |  | ATHUBA 2145 |
|  |  | ATHUBA 2146 |
|  |  | ATHUBA 2147 |
|  |  | ATHUBA 2148 |
|  |  | ATHUBA 2149 |
|  |  | ATHUBA 2150 |
| Crete | Rhizosphere *Salvia officinalis* | ATHUBA 1927 |
|  |  | ATHUBA 1937 |
|  |  | ATHUBA 1938 |
|  |  | ATHUBA 1940 |
|  |  | ATHUBA 1941 |
|  |  | ATHUBA 1942 |
|  |  | ATHUBA 1943 |
|  |  | ATHUBA 1944 |
|  |  | ATHUBA 1928 |
|  |  | ATHUBA 1946 |
|  |  | ATHUBA 1947 |
|  |  | ATHUBA 1948 |
|  |  | ATHUBA 1949 |
|  |  | ATHUBA 1950 |
|  |  | ATHUBA 1952 |
|  |  | ATHUBA 1954 |
|  |  | ATHUBA 1929 |
|  |  | ATHUBA 1930 |
|  |  | ATHUBA 1931 |
|  |  | ATHUBA 1932 |
|  |  | ATHUBA 1933 |
|  |  | ATHUBA 1934 |
|  |  | ATHUBA 1935 |
| Crete | Rhizosphere *Foeniculum vulgare* | ATHUBA 1955 |
|  |  | ATHUBA 1965 |
|  |  | ATHUBA 1967 |
|  |  | ATHUBA 1970 |
|  |  | ATHUBA 1957 |
|  |  | ATHUBA 1958 |
|  |  | ATHUBA 1960 |
| Crete | Rhizosphere *Rosmarinus officinalis* | ATHUBA 1972 |
|  |  | ATHUBA 1981 |
|  |  | ATHUBA 1984 |
|  |  | ATHUBA 1986 |
|  |  | ATHUBA 1987 |
|  |  | ATHUBA 1988 |
|  |  | ATHUBA 1990 |
|  |  | ATHUBA 1973 |
|  |  | ATHUBA 1991 |
|  |  | ATHUBA 1992 |
|  |  | ATHUBA 1993 |
|  |  | ATHUBA 1994 |
|  |  | ATHUBA 1995 |
|  |  | ATHUBA 1974 |
|  |  | ATHUBA 1975 |
|  |  | ATHUBA 1976 |
|  |  | ATHUBA 1977 |
|  |  | ATHUBA 1978 |
|  |  | ATHUBA 1980 |
| Crete | Rhizosphere *Olea europaea* | ATHUBA 1996 |
|  |  | ATHUBA 2006 |
|  |  | ATHUBA 2007 |
|  |  | ATHUBA 2008 |
|  |  | ATHUBA 2009 |
|  |  | ATHUBA 2010 |
|  |  | ATHUBA 2012 |
|  |  | ATHUBA 1997 |
|  |  | ATHUBA 2000 |
|  |  | ATHUBA 2001 |
|  |  | ATHUBA 2002 |
|  |  | ATHUBA 2003 |
|  |  | ATHUBA 2004 |
| Crete | Rhizosphere *Pinus brutia* | ATHUBA 2013 |
|  |  | ATHUBA 2014 |
| Crete | Rhizosphere *Pittosporum tobira* | ATHUBA 2051 |
|  |  | ATHUBA 2060 |
|  |  | ATHUBA 2061 |
|  |  | ATHUBA 2062 |
|  |  | ATHUBA 2065 |
|  |  | ATHUBA 2066 |
|  |  | ATHUBA 2067 |
|  |  | ATHUBA 2068 |
|  |  | ATHUBA 2052 |
|  |  | ATHUBA 2072 |
|  |  | ATHUBA 2073 |
|  |  | ATHUBA 2074 |
|  |  | ATHUBA 2076 |
|  |  | ATHUBA 2078 |
|  |  | ATHUBA 2079 |
|  |  | ATHUBA 2053 |
|  |  | ATHUBA 2080 |
|  |  | ATHUBA 2082 |
|  |  | ATHUBA 2083 |
|  |  | ATHUBA 2084 |
|  |  | ATHUBA 2085 |
|  |  | ATHUBA 2086 |
|  |  | ATHUBA 2087 |
|  |  | ATHUBA 2054 |
|  |  | ATHUBA 2090 |
|  |  | ATHUBA 2055 |
|  |  | ATHUBA 2056 |
|  |  | ATHUBA 2057 |
|  |  | ATHUBA 2058 |
|  |  | ATHUBA 2059 |
| Crete | Rhizosphere *Diospyros kaki* | ATHUBA 2101 |
|  |  | ATHUBA 2102 |
|  |  | ATHUBA 2103 |
|  |  | ATHUBA 2109 |
|  |  | ATHUBA 2111 |
|  |  | ATHUBA 2112 |
|  |  | ATHUBA 2113 |
|  |  | ATHUBA 2116 |
|  |  | ATHUBA 2117 |
|  |  | ATHUBA 2118 |
|  |  | ATHUBA 2119 |
|  |  | ATHUBA 2122 |
|  |  | ATHUBA 2123 |
|  |  | ATHUBA 2096 |
|  |  | ATHUBA 2097 |
|  |  | ATHUBA 2099 |
| Athens National Garden | Rhizosphere *Livistonia* sp. | ATHUBA 2518 |
|  |  | ATHUBA 2527 |
|  |  | ATHUBA 2528 |
|  |  | ATHUBA 2529 |
|  |  | ATHUBA 2530 |
|  |  | ATHUBA 2519 |
|  |  | ATHUBA 2520 |
|  |  | ATHUBA 2521 |
|  |  | ATHUBA 2523 |
|  |  | ATHUBA 2524 |
|  |  | ATHUBA 2525 |
|  |  | ATHUBA 2526 |
| Marathon | Soil | ATHUBA 246 |
|  |  | ATHUBA 255 |
|  |  | ATHUBA 345 |
|  |  | ATHUBA 347 |
|  |  | ATHUBA 348 |
|  |  | ATHUBA 350 |
|  |  | ATHUBA 351 |
|  |  | ATHUBA 352 |
|  |  | ATHUBA 256 |
|  |  | ATHUBA 353 |
|  |  | ATHUBA 281 |
|  |  | ATHUBA 354 |
|  |  | ATHUBA 284 |
|  |  | ATHUBA 285 |
|  |  | ATHUBA 768 |
|  |  | ATHUBA 294 |
|  |  | ATHUBA 298 |
|  |  | ATHUBA 299 |
|  |  | ATHUBA 300 |
|  |  | ATHUBA 257 |
|  |  | ATHUBA 355 |
|  |  | ATHUBA 356 |
|  |  | ATHUBA 357 |
|  |  | ATHUBA 303 |
|  |  | ATHUBA 358 |
|  |  | ATHUBA 323 |
|  |  | ATHUBA 330 |
|  |  | ATHUBA 258 |
|  |  | ATHUBA 259 |
|  |  | ATHUBA 261 |
|  |  | ATHUBA 262 |
|  |  | ATHUBA 263 |
|  |  | ATHUBA 264 |
|  |  | ATHUBA 247 |
|  |  | ATHUBA 265 |
|  |  | ATHUBA 266 |
|  |  | ATHUBA 267 |
|  |  | ATHUBA 268 |
|  |  | ATHUBA 269 |
|  |  | ATHUBA 272 |
|  |  | ATHUBA 273 |
|  |  | ATHUBA 274 |
|  |  | ATHUBA 275 |
|  |  | ATHUBA 276 |
|  |  | ATHUBA 248 |
|  |  | ATHUBA 277 |
|  |  | ATHUBA 278 |
|  |  | ATHUBA 279 |
|  |  | ATHUBA 280 |
|  |  | ATHUBA 282 |
|  |  | ATHUBA 283 |
|  |  | ATHUBA 287 |
|  |  | ATHUBA 288 |
|  |  | ATHUBA 289 |
|  |  | ATHUBA 290 |
|  |  | ATHUBA 291 |
|  |  | ATHUBA 292 |
|  |  | ATHUBA 293 |
|  |  | ATHUBA 295 |
|  |  | ATHUBA 296 |
|  |  | ATHUBA 297 |
|  |  | ATHUBA 250 |
|  |  | ATHUBA 301 |
|  |  | ATHUBA 302 |
|  |  | ATHUBA 304 |
|  |  | ATHUBA 305 |
|  |  | ATHUBA 306 |
|  |  | ATHUBA 307 |
|  |  | ATHUBA 249 |
|  |  | ATHUBA 251 |
|  |  | ATHUBA 308 |
|  |  | ATHUBA 309 |
|  |  | ATHUBA 310 |
|  |  | ATHUBA 311 |
|  |  | ATHUBA 312 |
|  |  | ATHUBA 313 |
|  |  | ATHUBA 314 |
|  |  | ATHUBA 315 |
|  |  | ATHUBA 316 |
|  |  | ATHUBA 252 |
|  |  | ATHUBA 317 |
|  |  | ATHUBA 318 |
|  |  | ATHUBA 319 |
|  |  | ATHUBA 320 |
|  |  | ATHUBA 321 |
|  |  | ATHUBA 322 |
|  |  | ATHUBA 324 |
|  |  | ATHUBA 325 |
|  |  | ATHUBA 253 |
|  |  | ATHUBA 327 |
|  |  | ATHUBA 328 |
|  |  | ATHUBA 329 |
|  |  | ATHUBA 331 |
|  |  | ATHUBA 332 |
|  |  | ATHUBA 333 |
|  |  | ATHUBA 335 |
|  |  | ATHUBA 254 |
|  |  | ATHUBA 336 |
|  |  | ATHUBA 338 |
|  |  | ATHUBA 339 |
|  |  | ATHUBA 340 |
|  |  | ATHUBA 342 |
|  |  | ATHUBA 344 |
| Athens National Garden | Leaves *Maclura* sp. | ATHUBA 1001 |
|  |  | ATHUBA 1002 |
|  |  | ATHUBA 1003 |
|  |  | ATHUBA 1005 |
| Oeta | Rhizosphere *Veronica oetaea* | ATHUBA 464 |
|  |  | ATHUBA 468 |
|  |  | ATHUBA 471 |
|  |  | ATHUBA 456 |
|  |  | ATHUBA 475 |
|  |  | ATHUBA 479 |
|  |  | ATHUBA 481 |
|  |  | ATHUBA 482 |
|  |  | ATHUBA 487 |
|  |  | ATHUBA 489 |
|  |  | ATHUBA 462 |
| Athens National Garden | Rhizosphere *Olea europa* | ATHUBA 915 |
|  |  | ATHUBA 907 |
|  |  | ATHUBA 908 |
|  |  | ATHUBA 909 |
|  |  | ATHUBA 910 |
|  |  | ATHUBA 911 |
|  |  | ATHUBA 913 |
|  |  | ATHUBA 914 |
| Crete | Rhizosphere *Olea europa* | ATHUBA 495 |
|  |  | ATHUBA 505 |
|  |  | ATHUBA 510 |
|  |  | ATHUBA 511 |
|  |  | ATHUBA 514 |
|  |  | ATHUBA 496 |
|  |  | ATHUBA 515 |
|  |  | ATHUBA 516 |
|  |  | ATHUBA 518 |
|  |  | ATHUBA 519 |
|  |  | ATHUBA 520 |
|  |  | ATHUBA 524 |
|  |  | ATHUBA 525 |
|  |  | ATHUBA 526 |
|  |  | ATHUBA 579 |
|  |  | ATHUBA 580 |
|  |  | ATHUBA 530 |
|  |  | ATHUBA 533 |
|  |  | ATHUBA 538 |
|  |  | ATHUBA 497 |
|  |  | ATHUBA 541 |
|  |  | ATHUBA 542 |
|  |  | ATHUBA 544 |
|  |  | ATHUBA 545 |
|  |  | ATHUBA 546 |
|  |  | ATHUBA 498 |
|  |  | ATHUBA 548 |
|  |  | ATHUBA 549 |
|  |  | ATHUBA 581 |
|  |  | ATHUBA 552 |
|  |  | ATHUBA 553 |
|  |  | ATHUBA 554 |
|  |  | ATHUBA 499 |
|  |  | ATHUBA 559 |
|  |  | ATHUBA 561 |
|  |  | ATHUBA 563 |
|  |  | ATHUBA 500 |
|  |  | ATHUBA 566 |
|  |  | ATHUBA 567 |
|  |  | ATHUBA 568 |
|  |  | ATHUBA 569 |
|  |  | ATHUBA 571 |
|  |  | ATHUBA 573 |
|  |  | ATHUBA 576 |
|  |  | ATHUBA 503 |
|  |  | ATHUBA 577 |
|  |  | ATHUBA 578 |
|  |  | ATHUBA 578 |
|  |  | ATHUBA 504 |
| Elefsina | Polluted soil | ATHUBA 739 |
|  |  | ATHUBA 740 |
|  |  | ATHUBA 741 |
| Athens National Garden | Rhizosphere *Querqus* sp. | ATHUBA 1015 |
|  |  | ATHUBA 1016 |
|  |  | ATHUBA 1019 |
|  |  | ATHUBA 1021 |
|  |  | ATHUBA 1022 |
|  |  | ATHUBA 1023 |
|  |  | ATHUBA 1025 |
|  |  | ATHUBA 1026 |
|  |  | ATHUBA 1027 |
|  |  | ATHUBA 1028 |
|  |  | ATHUBA 1029 |
|  |  | ATHUBA 1030 |
|  |  | ATHUBA 1031 |
|  |  | ATHUBA 1033 |
|  |  | ATHUBA 1036 |
|  |  | ATHUBA 1037 |
|  |  | ATHUBA 1038 |
|  |  | ATHUBA 1039 |
|  |  | ATHUBA 1040 |
|  |  | ATHUBA 1041 |
|  |  | ATHUBA 1042 |
|  |  | ATHUBA 1043 |
|  |  | ATHUBA 1009 |
|  |  | ATHUBA 1009 |
|  |  | ATHUBA 1045 |
|  |  | ATHUBA 1048 |
|  |  | ATHUBA 1051 |
|  |  | ATHUBA 1053 |
|  |  | ATHUBA 1054 |
|  |  | ATHUBA 1010 |
|  |  | ATHUBA 1055 |
|  |  | ATHUBA 1060 |
|  |  | ATHUBA 1011 |
|  |  | ATHUBA 1012 |
|  |  | ATHUBA 1013 |
|  |  | ATHUBA 1014 |
| Athens National Garden | Rhizosphere *Rosa banksiae* | ATHUBA 868 |
|  |  | ATHUBA 877 |
|  |  | ATHUBA 878 |
|  |  | ATHUBA 879 |
|  |  | ATHUBA 880 |
|  |  | ATHUBA 881 |
|  |  | ATHUBA 882 |
|  |  | ATHUBA 883 |
|  |  | ATHUBA 884 |
|  |  | ATHUBA 869 |
|  |  | ATHUBA 870 |
|  |  | ATHUBA 871 |
|  |  | ATHUBA 872 |
|  |  | ATHUBA 873 |
|  |  | ATHUBA 874 |
|  |  | ATHUBA 875 |
|  |  | ATHUBA 876 |
| Kaisariani | Rhizosphere *Ebenus sipthorpii* | ATHUBA 195 |
|  |  | ATHUBA 196 |
|  |  | ATHUBA 198 |
|  |  | ATHUBA 197 |
|  |  | ATHUBA 199 |
|  |  | ATHUBA 202 |
|  |  | ATHUBA 203 |
|  |  | ATHUBA 204 |
|  |  | ATHUBA 209 |
|  |  | ATHUBA 208 |
|  |  | ATHUBA 192 |
|  |  | ATHUBA 193 |
|  |  | ATHUBA 194 |
|  |  | ATHUBA 200 |
|  |  | ATHUBA 201 |
|  |  | ATHUBA 205 |
|  |  | ATHUBA 206 |
| Marathon | Agricultural soil | ATHUBA 682 |
|  |  | ATHUBA 796 |
|  |  | ATHUBA 674 |
|  |  | ATHUBA 676 |
|  |  | ATHUBA 677 |
|  |  | ATHUBA 663 |
|  |  | ATHUBA 678 |
|  |  | ATHUBA 664 |
|  |  | ATHUBA 680 |
|  |  | ATHUBA 665 |
|  |  | ATHUBA 666 |
|  |  | ATHUBA 679 |
|  |  | ATHUBA 667 |
|  |  | ATHUBA 668 |
|  |  | ATHUBA 669 |
|  |  | ATHUBA 661 |
|  |  | ATHUBA 670 |
|  |  | ATHUBA 671 |
|  |  | ATHUBA 662 |
|  |  | ATHUBA 672 |
|  |  | ATHUBA 673 |
|  |  | ATHUBA 681 |
| Athens National Garden | Rhizosphere *Selaginella* | ATHUBA 999 |
|  |  | ATHUBA 996 |
| Athens National Garden | Rhizosphere *Tipuana tipu* | ATHUBA 916 |
|  |  | ATHUBA 925 |
|  |  | ATHUBA 926 |
|  |  | ATHUBA 917 |
|  |  | ATHUBA 918 |
|  |  | ATHUBA 920 |
|  |  | ATHUBA 922 |
|  |  | ATHUBA 923 |
|  |  | ATHUBA 924 |
| Thermopylae | Soil | ATHUBA 752 |
|  |  | ATHUBA 728 |
|  |  | ATHUBA 730 |
|  |  | ATHUBA 721 |
|  |  | ATHUBA 723 |
|  |  | ATHUBA 751 |
|  |  | ATHUBA 752 |
| Santorini | Volcanic sediment | ATHUBA 749 |
| Athens National Garden | Rhizosphere *Washingtonia* sp. | ATHUBA 972 |
|  |  | ATHUBA 973 |
|  |  | ATHUBA 974 |
|  |  | ATHUBA 975 |
|  |  | ATHUBA 979 |
|  |  | ATHUBA 964 |
|  |  | ATHUBA 982 |
|  |  | ATHUBA 984 |
|  |  | ATHUBA 985 |
|  |  | ATHUBA 965 |
|  |  | ATHUBA 987 |
|  |  | ATHUBA 988 |
|  |  | ATHUBA 989 |
|  |  | ATHUBA 966 |
|  |  | ATHUBA 991 |
|  |  | ATHUBA 992 |
|  |  | ATHUBA 967 |
|  |  | ATHUBA 968 |
|  |  | ATHUBA 969 |
|  |  | ATHUBA 970 |
|  |  | ATHUBA 971 |
| Athens National Garden | Rhizosphere *Wisteria* sp. | ATHUBA 959 |
|  |  | ATHUBA 961 |
|  |  | ATHUBA 962 |
|  |  | ATHUBA 951 |
|  |  | ATHUBA 952 |
|  |  | ATHUBA 953 |
|  |  | ATHUBA 954 |
|  |  | ATHUBA 955 |
|  |  | ATHUBA 956 |
|  |  | ATHUBA 957 |
| Crete | Rhizosphere *Ceratonia siliqua* | ATHUBA 190 |
|  |  | ATHUBA 191 |
|  |  | ATHUBA 189 |
| Hymettus | Soil | ATHUBA 1376 |
|  |  | ATHUBA 1388 |
|  |  | ATHUBA 1373 |
| Hymettus | Rhizosphere *Cupressus* sp. | ATHUBA 1398 |
|  |  | ATHUBA 1400 |
|  |  | ATHUBA 1401 |
|  |  | ATHUBA 1390 |
|  |  | ATHUBA 1391 |
|  |  | ATHUBA 1392 |
|  |  | ATHUBA 1394 |
|  |  | ATHUBA 1395 |
| Hymettus | Rhizosphere *Selaginella* | ATHUBA 1402 |
|  |  | ATHUBA 1404 |
|  |  | ATHUBA 1406 |
|  |  | ATHUBA 1407 |
| Hymettus | Soil | ATHUBA 1419 |
|  |  | ATHUBA 1413 |
|  |  | ATHUBA 1414 |
|  |  | ATHUBA 1415 |
|  |  | ATHUBA 1416 |
|  |  | ATHUBA 1418 |
|  |  | ATHUBA 1421 |
|  |  | ATHUBA 1419 |
|  |  | ATHUBA 1425 |
|  |  | ATHUBA 1426 |
| Hymettus | Rhizosphere *Ceratonia siliqua* | ATHUBA 1471 |
|  |  | ATHUBA 1479 |
|  |  | ATHUBA 1480 |
|  |  | ATHUBA 1482 |
|  |  | ATHUBA 1483 |
|  |  | ATHUBA 1484 |
|  |  | ATHUBA 1485 |
|  |  | ATHUBA 1486 |
|  |  | ATHUBA 1472 |
|  |  | ATHUBA 1489 |
|  |  | ATHUBA 1490 |
|  |  | ATHUBA 1493 |
|  |  | ATHUBA 1494 |
|  |  | ATHUBA 1474 |
|  |  | ATHUBA 1477 |
| Hymettus | Rhizosphere *Nerium oleander* | ATHUBA 1495 |
|  |  | ATHUBA 1510 |
|  |  | ATHUBA 1511 |
|  |  | ATHUBA 1497 |
|  |  | ATHUBA 1498 |
|  |  | ATHUBA 1499 |
| Hymettus | Rhizosphere *Pyracantha* sp. | ATHUBA 1512 |
|  |  | ATHUBA 1521 |
|  |  | ATHUBA 1523 |
|  |  | ATHUBA 1524 |
|  |  | ATHUBA 1526 |
|  |  | ATHUBA 1527 |
|  |  | ATHUBA 1528 |
|  |  | ATHUBA 1530 |
|  |  | ATHUBA 1531 |
|  |  | ATHUBA 1534 |
|  |  | ATHUBA 1535 |
|  |  | ATHUBA 1537 |
|  |  | ATHUBA 1514 |
|  |  | ATHUBA 1515 |
|  |  | ATHUBA 1517 |
|  |  | ATHUBA 1519 |
| Hymettus | Rhizosphere *Pyracantha* sp. | ATHUBA 1549 |
|  |  | ATHUBA 1550 |
|  |  | ATHUBA 1551 |
|  |  | ATHUBA 1552 |
|  |  | ATHUBA 1554 |
|  |  | ATHUBA 1555 |
|  |  | ATHUBA 1556 |
|  |  | ATHUBA 1558 |
|  |  | ATHUBA 1542 |
|  |  | ATHUBA 1543 |
|  |  | ATHUBA 1545 |
|  |  | ATHUBA 1546 |
|  |  | ATHUBA 1547 |
| Hymettus | Rhizosphere *Quercus coccifera* | ATHUBA 1303 |
|  |  | ATHUBA 1304 |
|  |  | ATHUBA 1305 |
|  |  | ATHUBA 1306 |
|  |  | ATHUBA 1307 |
|  |  | ATHUBA 1308 |
|  |  | ATHUBA 1309 |
|  |  | ATHUBA 1310 |
|  |  | ATHUBA 1311 |
|  |  | ATHUBA 1315 |
|  |  | ATHUBA 1322 |
|  |  | ATHUBA 1296 |
|  |  | ATHUBA 1298 |
|  |  | ATHUBA 1300 |
|  |  | ATHUBA 1302 |
| Zagorochoria | Rhizosphere *Quercus coccifera* | ATHUBA 1754 |
|  |  | ATHUBA 1763 |
|  |  | ATHUBA 1764 |
|  |  | ATHUBA 1765 |
|  |  | ATHUBA 1766 |
|  |  | ATHUBA 1767 |
|  |  | ATHUBA 1768 |
|  |  | ATHUBA 1770 |
|  |  | ATHUBA 1771 |
|  |  | ATHUBA 1774 |
|  |  | ATHUBA 1775 |
|  |  | ATHUBA 1777 |
|  |  | ATHUBA 1780 |
|  |  | ATHUBA 1781 |
|  |  | ATHUBA 1782 |
|  |  | ATHUBA 1756 |
|  |  | ATHUBA 1783 |
|  |  | ATHUBA 1784 |
|  |  | ATHUBA 1786 |
|  |  | ATHUBA 1787 |
|  |  | ATHUBA 1788 |
|  |  | ATHUBA 1789 |
|  |  | ATHUBA 1757 |
|  |  | ATHUBA 1759 |
|  |  | ATHUBA 1761 |
| Zagorochoria | Rhizosphere *Cedrus libani* | ATHUBA 1806 |
|  |  | ATHUBA 1815 |
|  |  | ATHUBA 1819 |
|  |  | ATHUBA 1820 |
|  |  | ATHUBA 1821 |
|  |  | ATHUBA 1822 |
|  |  | ATHUBA 1824 |
|  |  | ATHUBA 1825 |
|  |  | ATHUBA 1826 |
|  |  | ATHUBA 1827 |
|  |  | ATHUBA 1810 |
|  |  | ATHUBA 1812 |
|  |  | ATHUBA 1814 |
| Crete | Rhizosphere *Olea europa* | ATHUBA 582 |
|  |  | ATHUBA 584 |
|  |  | ATHUBA 780 |
|  |  | ATHUBA 586 |
|  |  | ATHUBA 585 |
| Rhodes | Marine sediment | ATHUBA 800 |

**Supplementary Table 2:** Frequency of use of liquid media in recent literature for the production of secondary metabolites. (References listed at the end of this file.)

| Medium name | Number of publications | References |
| --- | --- | --- |
| ISP2 (Yeast Extract, Malt Extract) | 24 | (Alhadrami et al, 2021; Ayswarya, 2020; Benreguieg, 2017; Fang et al, 2018; Ganesan, 2016; Gurovic & Olivera, 2017; Hamed, 2017; Hou et al, 2021; Jacob et al, 2017; Kaari et al, 2022; Kurnianto et al, 2020; Li et al, 2019; Lim, 2018; Mohamed et al, 2017; Nandhini, 2018; Phongsopitanun, 2020; Reegan et al, 2021; Ribeiro et al, 2020; Shrestha et al, 2021; Siddharth et al, 2020; Sproule et al, 2019; Wang et al, 2020; Wang et al, 2019; Wu et al, 2018) |
| SCB (Starch Casein Broth) | 9 | (Alghamdi, 2021; Bhosale et al, 2018; Bommareddy, 2018; Gomathi & Gothandam, 2019; Jacob et al, 2017; Nandhini, 2018; Odumosu et al, 2017; Pavan Kumar et al, 2018; Sapkota et al, 2020) |
| MB (Marine Broth) | 8 | (Chen et al, 2018a; Fang et al, 2020; Gozari et al, 2018; le Roes-Hill et al, 2018; Paderog et al, 2020; Quintero et al, 2018; Ribeiro et al, 2020; Wibowo et al, 2019) |
| FEM (Fermentation medium) | 8 | (Ahmed et al, 2020; Cao, 2019; Jiang et al, 2018a; Meng-Xi et al, 2021; Pachaiyappan, 2017; Qi et al, 2019; Somasundaram, 2020; Yang et al, 2019) |
| TSB (Tryptone Soy Broth) | 7 | (Chemoh et al, 2021; Khadayat et al, 2020; Kim et al, 2020; Lotfy et al, 2019; Quintero et al, 2018; Uttara, 2020; Wu et al, 2018) |
| ISP4 (inorganic salts-starch) | 6 | (Ahmad et al, 2017; Jacob et al, 2017; Lotfy et al, 2019; Masand et al, 2018; Sebak et al, 2019; Wu et al, 2018) |
| ISP1 (tryptone yeast extract) | 6 | (Dhaneesha et al, 2017; Jacob et al, 2017; Krishnan, 2018; Manimaran et al, 2018; Ravi & Kannabiran, 2018; Ravi, 2017) |
| Μ1 | 4 | (Chen et al, 2019; Chen, 2021; Fatin et al, 2017; Jose & Jha, 2017) |
| SCN (Starch Casein Nitrate) | 3 | (Ashraf et al, 2021; Sharma & Manhas, 2019; Sharma, 2021) |
| NB (Nutrient broth) | 4 | (Jacob et al, 2017; Kurnianto et al, 2020; Mahfouz, 2021; Sajjad et al, 2018) |
| GYM (Glucose, Yeast Extract, Malt Extract) | 3 | (Aslam, 2020; Fatima et al, 2019; Fatima, 2018) |
| HFM1 (Han’s Fermentation Media 1) | 3 | (Kemung et al, 2020; Law et al, 2019; Mangzira Kemung et al, 2020) |
| APM (Antibiotic production medium) | 3 | (Lim, 2018; Pachaiyappan, 2017; Sundaramanickam, 2019) |
| GYM4 | 2 | (Arn et al, 2020; Peng et al, 2020) |
| GLM- Glucose yeast extract malt medium | 2 | (Mehetre et al, 2019; Pachaiyappan, 2017) |
| M3 media- Micromonospora medium | 2 | (Ganesan, 2016; Pachaiyappan, 2017) |
| YPG (Yeast peptone glucose medium) | 2 | (Ganesan, 2016; Pachaiyappan, 2017) |
| A1 | 2 | (Hou et al, 2021; Quintero et al, 2018) |
| PDB (Potato Dextrose Broth) | 2 | (Bundale et al, 2019; Sharma et al, 2021) |
| Seawater-Based Fermentation Medium | 2 | (Dhaneesha et al, 2017; Wu et al, 2017) |
| YIM 38 | 1 | (Jiang et al, 2018b) |
| CYPS | 1 | (Shah et al, 2017) |
| GSB (Glucose soybean meal broth) | 1 | (Jacob et al, 2017) |
| MNG | 1 | (Nithya et al, 2018) |
| Gauserime 1 broth | 1 | (Chen et al, 2018b) |
| X-medium | 1 | (Singh et al, 2018) |
| ACM | 1 | (Wu et al, 2018) |
| CQ-1 | 1 | (Wu et al, 2018) |
| SAO-23 | 1 | (Wu et al, 2018) |
| FP-1 | 1 | (Wu et al, 2018) |
| NL200 | 1 | (Nafis et al, 2018) |
| GSS | 1 | (Liu et al, 2019) |
| R5A | 1 | (Malmierca et al, 2018) |
| Gause’s liquid | 1 | (Zhou et al, 2019) |
| SS (soluble starch) | 1 | (Kamarudheen & Rao, 2019) |
| SYP-SW liquid | 1 | (Choi et al, 2019) |
| SPM1 | 1 | (Peng et al, 2020) |
| R2A | 1 | (Arn et al, 2020) |
| NL148sb | 1 | (Arn et al, 2020) |
| MR5 | 1 | (Leimer et al, 2021) |
| ISP3 | 1 | (Lim, 2018) |
| Kuster's broth | 1 | (Jacob et al, 2017) |
| Sabouraud dextrose broth | 1 | (Jacob et al, 2017) |
| Gause synthetic broth (GSB) | 1 | (Kurnianto et al, 2020) |
| AM2-ab | 1 | (Pachaiyappan, 2020) |
| R2YE | 1 | (Katif, 2022) |
| MNGA | 1 | (Ganesan, 2016) |
| BENNET | 1 | (Ganesan, 2016) |
| Koch 1 liquid | 1 | (Zhao et al, 2020) |
| Medium Bran | 1 | (Zhao et al, 2020) |
| A1BFe+C | 1 | (Norouzi et al, 2018) |
| DSM67 | 1 | (Hou et al, 2021) |
| PYG | 1 | (Hou et al, 2021) |
| Liquid A | 1 | (Zhang et al, 2021) |
| YMG (Yeast malt glucose) | 1 | (Sharma, 2022) |
| SM2 (Synthetic medium 2) | 1 | (Sharma, 2022) |
| A-medium | 1 | (Cheema et al, 2021) |
| MNG (Modified nutrient glucose medium) | 1 | (Pachaiyappan, 2017) |
| M6 medium | 1 | (Pachaiyappan, 2017) |
| CYSP medium | 1 | (Mehetre et al, 2019) |
| MGYP medium | 1 | (Mehetre et al, 2019) |
| Medium 333 | 1 | (Mehetre et al, 2019) |
| Μedium 30 | 1 | (Kuncharoen, 2019) |
| SGG | 1 | (Handayani et al, 2021) |
| YM | 1 | (Handayani et al, 2021) |
| OM | 1 | (Handayani et al, 2021) |
| R5 | 1 | (Handayani et al, 2021) |
| MS | 1 | (Handayani et al, 2021) |
| TSG | 1 | (Handayani et al, 2021) |
| NL19 | 1 | (Handayani et al, 2021) |
| NL300 | 1 | (Handayani et al, 2021) |
| NL330 | 1 | (Handayani et al, 2021) |
| NL500 | 1 | (Handayani et al, 2021) |
| NL550 | 1 | (Handayani et al, 2021) |
| NL800 | 1 | (Handayani et al, 2021) |

**Supplementary Table 3.** Screening results of the top 91 selected extracts. All extracts exhibit high tyrosinase and/or elastase inhibition activities in cell-free *in vitro assays*. Extracts that cause <90% cell viability up to 10 μg/mL at 24h were marked as toxic. Non toxic and highly active extracts were chosen for cell-based testing.

| **Extract** | **Cell-free tyrosinase inhibition**  **%** | **SD %** | **Cell-free elastase inhibition %** | **SD %** | **Cytotoxicity 24h  (up to 10μg/mL)** |
| --- | --- | --- | --- | --- | --- |
| **292 E** | **87.6438** | 6.42072 | -9.1561 | 0.14674 | NON TOXIC |
| **376 E** | **85.0302** | 4.52064 | -24.808 | 2.00663 | NON TOXIC |
| **578 E** | **83.9321** | 14.8456 | -108.58 | 3.675 | NON TOXIC |
| **1526 M** | **83.545** | 0.40877 | -43.617 | 9.81464 | NON TOXIC |
| **728 M** | **83.0152** | 0.07281 | -36.929 | 35.147 | NON TOXIC |
| **204 M** | **82.522** | 10.14 | 8.415 | 5.057 | NON TOXIC |
| **395 E** | **82.1828** | 5.97245 | -26.293 | 3.60404 | NON TOXIC |
| **1494 E** | **77.14** | 3.58862 | -38.434 | 3.13961 | NON TOXIC |
| **1388 E** | **76.6828** | 6.78444 | -41.962 | 2.2366 | NON TOXIC |
| **351 E** | **72.1759** | 2.07578 | -9.9744 | 0.91368 | NON TOXIC |
| **546 E** | **71.3371** | 5.24472 | -24.781 | 4.52334 | NON TOXIC |
| **1011 E** | **70.389** | 4.331 | **23.0305** | 1.94819 | NON TOXIC |
| **553 E** | **69.6049** | 2.02246 | -20.146 | 0.79848 | NON TOXIC |
| **361 M** | **67.739** | 6.577 | -31.932 | 0.09166 | NON TOXIC |
| **274 E** | **66.588** | 4.056 | -5.2116 | 1.41135 | NON TOXIC |
| **134 M** | **58.845** | 0.712 | -46.4 | 1.07604 | NON TOXIC |
| **664 M** | **58.7237** | 2.72417 | -128.17 | 11.93 | NON TOXIC |
| **1872 M** | **58.4282** | 1.95874 | -13.078 | 19.5289 | NON TOXIC |
| **2155 E** | **56.5516** | 0.39386 | -54.858 | 0.87407 | NON TOXIC |
| **1960 M** | **52.2118** | 2.62265 | -41.787 | 7.81397 | NON TOXIC |
| **908 M** | **49.4714** | 4.1515 | -3.913 | 0.194 | NON TOXIC |
| **336 E** | **31.5354** | 1.90131 | **64.708** | 1.75489 | NON TOXIC |
| **455 M** | **30.7189** | 2.3231 | 10.456 | 8.15186 | NON TOXIC |
| **134 E** | **30.538** | 12.071 | -9.0196 | 9.76642 | NON TOXIC |
| **481 M** | **28.9185** | 9.51945 | -27.739 | 4.80277 | NON TOXIC |
| **379 E** | **26.4492** | 7.11097 | -25.761 | 2.18901 | NON TOXIC |
| **332 E** | 5.54708 | 1.13997 | **57.4284** | 8.7247 | NON TOXIC |
| **284 E** | -2.2163 | 2.85584 | **60.8092** | 2.77968 | NON TOXIC |
| **325 E** | -20.721 | 2.999 | **29.2035** | 10.9495 | NON TOXIC |
| 340 E | 103.492 | 0.11873 | -72.865 | 0.35754 | TOXIC |
| 567 E | 94.6211 | 4.0313 | -9.6935 | 4.54661 | TOXIC |
| 953 M | 91.565 | 3.94539 | -41.969 | 4.95241 | TOXIC |
| 481 E | 89.4509 | 0.16715 | -55.58 | 2.05788 | TOXIC |
| 2156 E | 88.2215 | 0.16405 | -16.579 | 14.2399 | TOXIC |
| 1526 E | 85.3488 | 1.02133 | -56.881 | 8.00173 | TOXIC |
| 148 M | 84.4309 | 3.26805 | -114.91 | 7.905 | TOXIC |
| 970 E | 82.8507 | 1.56282 | -36.56 | 14.8046 | TOXIC |
| 573 E | 81.1486 | 0.85586 | -91.057 | 4.488 | TOXIC |
| 798 E | 80.472 | 16.759 | -107.49 | 13.6997 | TOXIC |
| 2130 E | 78.5418 | 6.55171 | -60.32 | 3.44066 | TOXIC |
| 755 E | 78.2381 | 2.42359 | -54.499 | 34.0998 | TOXIC |
| 214 E | 75.166 | 0.13429 | -8.3371 | 5.81557 | TOXIC |
| 2013 E | 74.9634 | 1.98755 | 2.64372 | 1.05747 | TOXIC |
| 242 E | 72.348 | 12.679 | -85.342 | 19.4254 | TOXIC |
| 2389 E | 68.2214 | 2.08112 | -121.2 | 34.449 | TOXIC |
| 392 E | 66.471 | 4.516 | -33.351 | 13.9531 | TOXIC |
| 387 E | 63.924 | 10.092 | -34.636 | 3.2395 | TOXIC |
| 158 M | 61.0238 | 1.95873 | -51.863 | 3.59476 | TOXIC |
| 350 E | 59.6746 | 1.66174 | -83.402 | 17.455 | TOXIC |
| 390 M | 58.9412 | 2.32569 | -56.59 | 1.22887 | TOXIC |
| 2524 M | 57.951 | 2.88746 | -2.454 | 1.149 | TOXIC |
| 2155 M | 57.2296 | 4.74266 | -97.189 | 1.59229 | TOXIC |
| 924 E | 56.3352 | 1.37866 | -12.418 | 14.6861 | TOXIC |
| 379 M | 56.232 | 27.894 | -49.132 | 5.62093 | TOXIC |
| 165 E | 54.7391 | 11.4295 | -107.42 | 15.8817 | TOXIC |
| 1861 E | 54.1466 | 0.76528 | -18.961 | 0.29838 | TOXIC |
| 2013 M | 52.3108 | 1.73462 | -96.577 | 1.50191 | TOXIC |
| 800 M | 51.8864 | 0.42427 | -23.043 | 16.687 | TOXIC |
| 213 E | 51.5308 | 10.5237 | -85.804 | 5.27476 | TOXIC |
| 2116 M | 50.3534 | 8.93119 | -100.95 | 8.77049 | TOXIC |
| 1949 M | 50.3236 | 1.241 | -64.724 | 6.12501 | TOXIC |
| 150 E | 50.2103 | 7.46234 | 15.8717 | 3.52614 | TOXIC |
| 392 M | 49.792 | 10.318 | -48.866 | 1.40567 | TOXIC |
| 123 E | 49.2689 | 5.49905 | -14.242 | 8.9242 | TOXIC |
| 2524 E | 48.3045 | 7.13657 | -27.364 | 1.90139 | TOXIC |
| 933 E | 48.0382 | 3.27114 | -35.531 | 3.65863 | TOXIC |
| 159 E | 46.8303 | 6.77061 | -26.728 | 6.2534 | TOXIC |
| 2564 E | 44.17 | 0.4139 | -19.94 | 7.07513 | TOXIC |
| 124 E | 43.885 | 8.505 | -104.7 | 11.3903 | TOXIC |
| 1875 M | 43.4935 | 0.90438 | 20.8216 | 2.3635 | TOXIC |
| 350 M | 41.9186 | 1.3038 | -33.021 | 12.0758 | TOXIC |
| 258 E | 38.037 | 6.7959 | -15.784 | 25.736 | TOXIC |
| 530 E | 37.8995 | 5.59557 | -17.941 | 8.6096 | TOXIC |
| 272 E | 35.7773 | 2.90955 | -7.2756 | 5.5281 | TOXIC |
| 2130 M | 31.7379 | 8.39879 | -75.96 | 7.68601 | TOXIC |
| 191 M | 31.134 | 1.758 | 21.798 | 0.68 | TOXIC |
| 744 E | 29.7892 | 6.78056 | -25.243 | 0.14848 | TOXIC |
| 361 E | 26.008 | 0.876 | -113.4 | 3.26806 | TOXIC |
| 545 E | 25.6474 | 7.18622 | 25.1309 | 1.18819 | TOXIC |
| 153 E | 25.4097 | 14.0278 | 31.997 | 1.931 | TOXIC |
| 1981 E | 13.7476 | 3.64894 | 45.478 | 0.76374 | TOXIC |
| 1043 E | 7.804 | 0.347 | 22.6869 | 11.0683 | TOXIC |
| 281 E | -1.8267 | 0.15784 | 23.1981 | 3.95611 | TOXIC |
| 329 E | -3.2 | 1.3 | 39.9477 | 0.19903 | TOXIC |
| 291 E | -9.4114 | 10.6923 | 29.681 | 4.393 | TOXIC |
| 215 E | -15.345 | 0.89 | 33.9897 | 0.53294 | TOXIC |
| 1037 E | -29.743 | 9.433 | 21.68 | 2.96239 | TOXIC |
| 1037 E | -29.743 | 9.433 | 21.68 | 2.96239 | TOXIC |
| 638 E | -33.514 | 7.01273 | 21.3368 | 0.24697 | TOXIC |
| 285 E | -52.973 | 23.8583 | 57.232 | 0.30431 | TOXIC |
| 330 E | -71.972 | 8.15288 | 25.575 | 1.118 | TOXIC |

**Supplementary Table 4.** Summary results of *in vitro* enzymatic inhibition (%) and cytotoxicity tests of the 14 ATHUBA 292 E fractions. Extracts that cause <90% cell viability up to 10 μg/mL at 24h were marked as toxic. All fractions were tested for their inhibition activity in cell-based assays.

|  | | ***In vitro* enzymatic inhibition** | | | | **Cytotoxicity 24h** | |
| --- | --- | --- | --- | --- | --- | --- | --- |
| **Extract** | **Fraction** | **Tyrosinase inhibition %** | **SD%** | **Elastase inhibition %** | **SD%** | **BJ** | **HaCaT** |
| **ATHUBA 292 E** | F1 | 8.386 | 9.808 | -0.379 | 7.467 | TOXIC | NON TOXIC |
|  | F2 | 0.272 | 17.437 | -8.308 | 6.630 | NON TOXIC | NON TOXIC |
|  | F3 | -44.016 | 10.834 | 3.416 | 10.857 | TOXIC | NON TOXIC |
|  | **F4** | -70.807 | 6.544 | **37.484** | 6.630 | NON TOXIC | NON TOXIC |
|  | **F5** | -29.238 | 6.988 | **47.957** | 12.738 | NON TOXIC | NON TOXIC |
|  | F6 | **55.213** | 10.257 | -10.259 | 4.804 | NON TOXIC | TOXIC |
|  | **F7** | -30.100 | 1.795 | **44.191** | 6.122 | NON TOXIC | NON TOXIC |
|  | F8 | -5.168 | 7.052 | **46.889** | 2.114 | TOXIC | NON TOXIC |
|  | **F9** | -29.329 | 10.706 | **43.822** | 5.545 | NON TOXIC | NON TOXIC |
|  | F10 | -3.400 | 8.911 | 4.131 | 0.686 | NON TOXIC | NON TOXIC |
|  | **F11** | -1.405 | 8.142 | **53.577** | 0.625 | NON TOXIC | NON TOXIC |
|  | **F12** | -27.153 | 8.398 | **57.750** | 15.167 | NON TOXIC | NON TOXIC |
|  | F13 | -31.097 | 2.949 | **34.728** | 2.539 | NON TOXIC | TOXIC |
|  | F14 | -47.779 | 2.564 | **41.357** | 6.616 | TOXIC | NON TOXIC |

**Supplementary Figure 1.** ^1^H NMR of Cyclo (L-proline-L-tyrosine) in CDCl_3_.


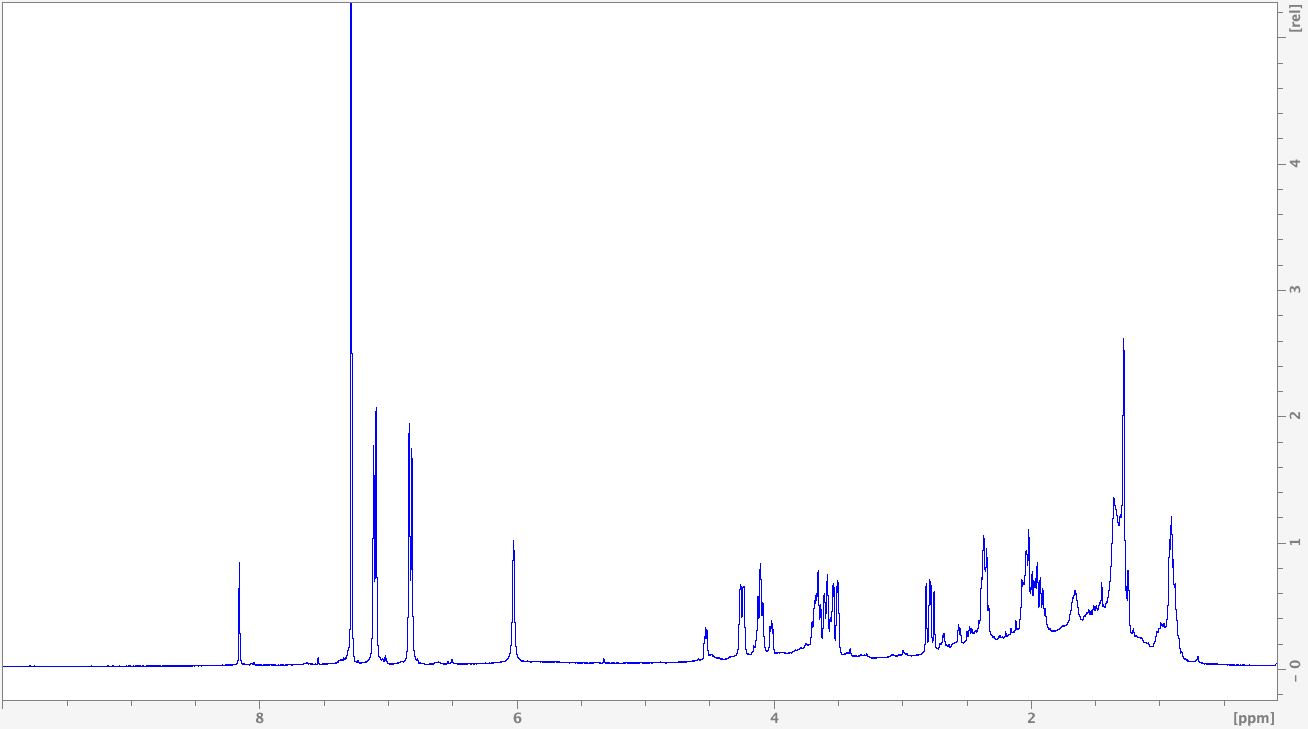


**Supplementary Figure 2.** ^1^H NMR of Cyclo (Pro-Phe) in CDCl_3_.

**
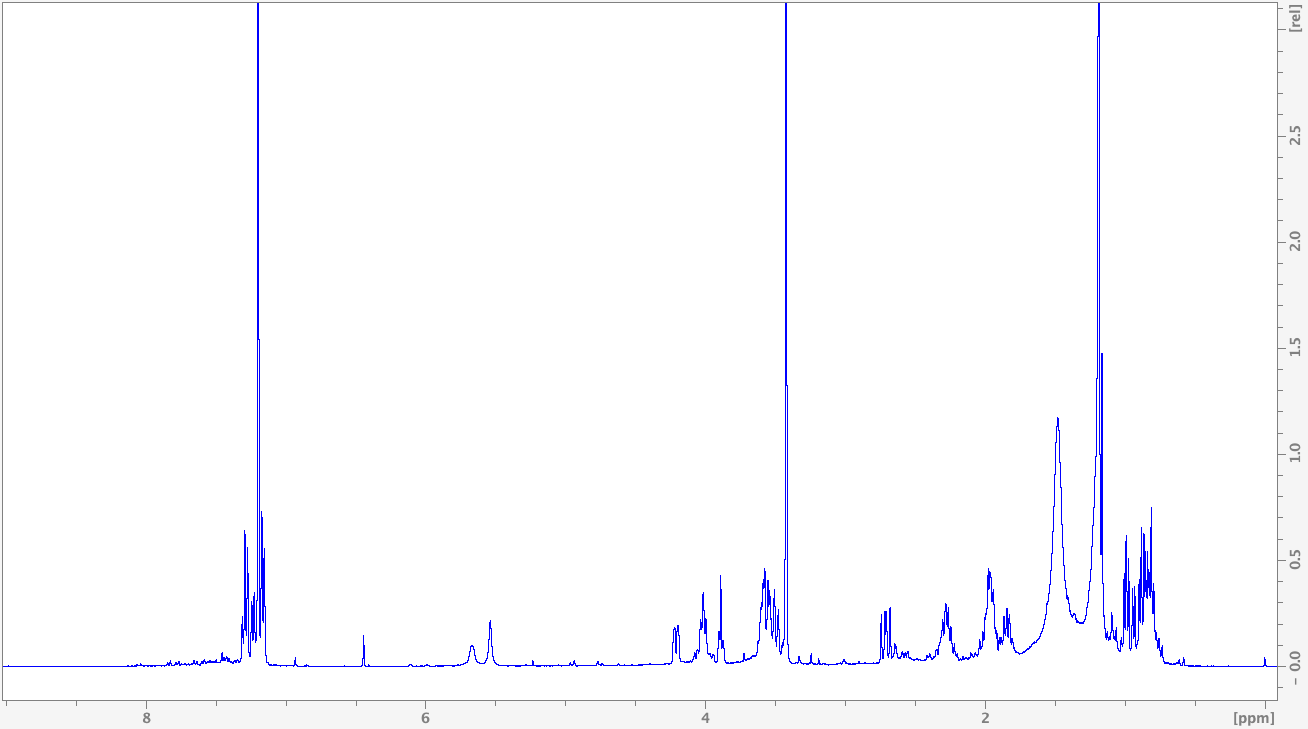
**

**Supplementary Figure 3.** ^1^H NMR of Lumichrome in MeOD-d4.

**
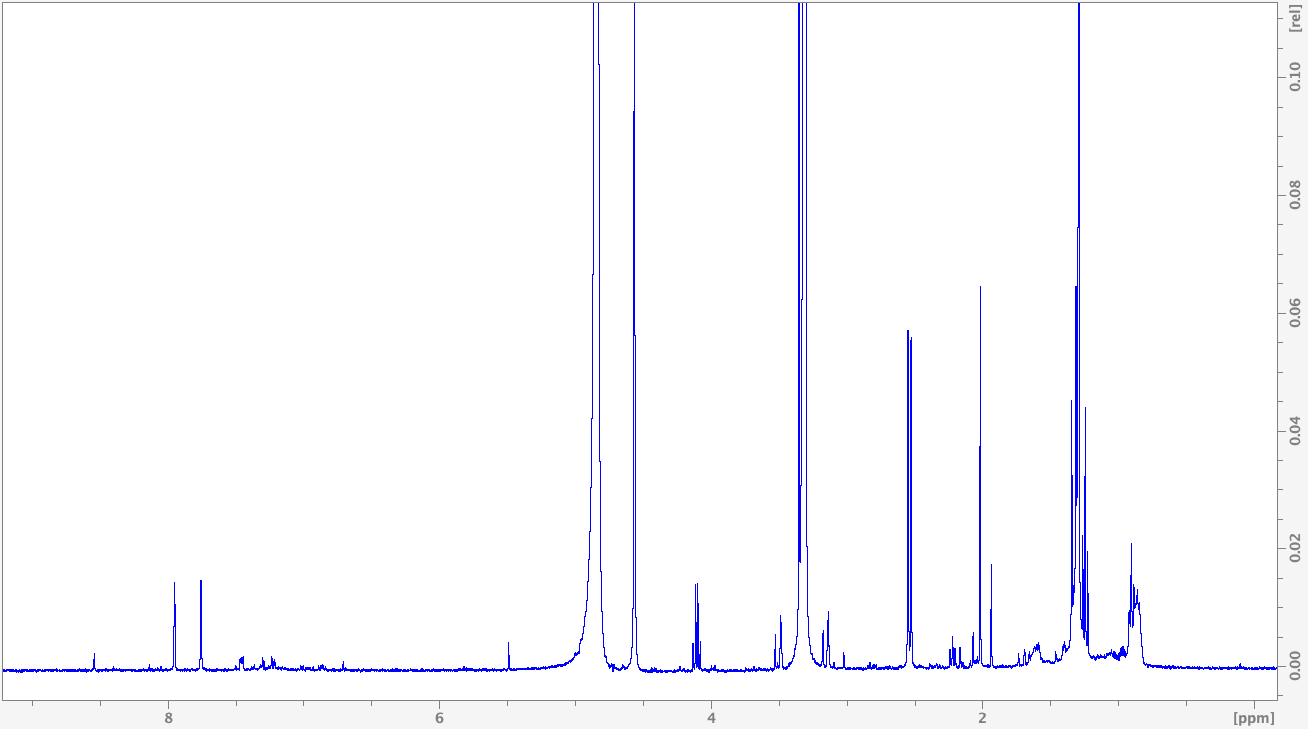
**

**Supplementary Figure 4.** ^1^H NMR of P-(acetylamino) benzoic in MeOD-d4.

**
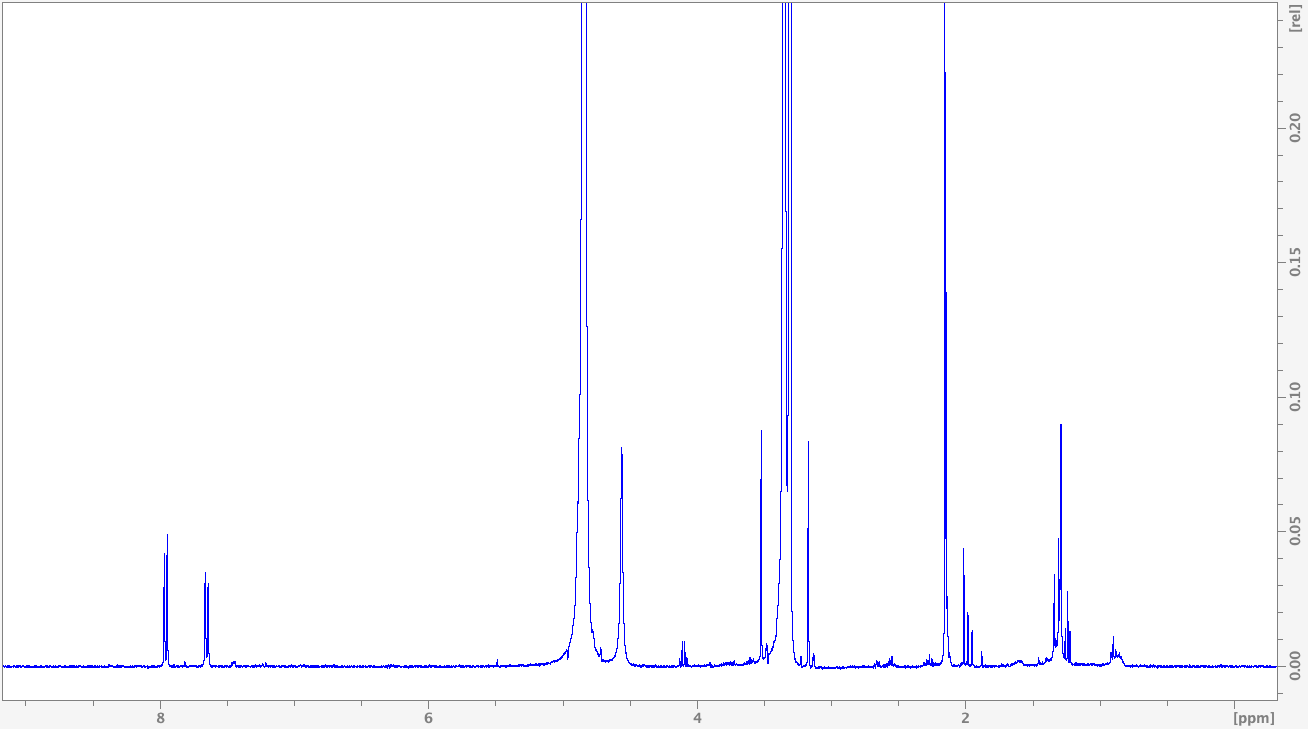
**

**Supplementary Figure 5.** ^1^H NMR of Daidzein in MeOD-d4.

**
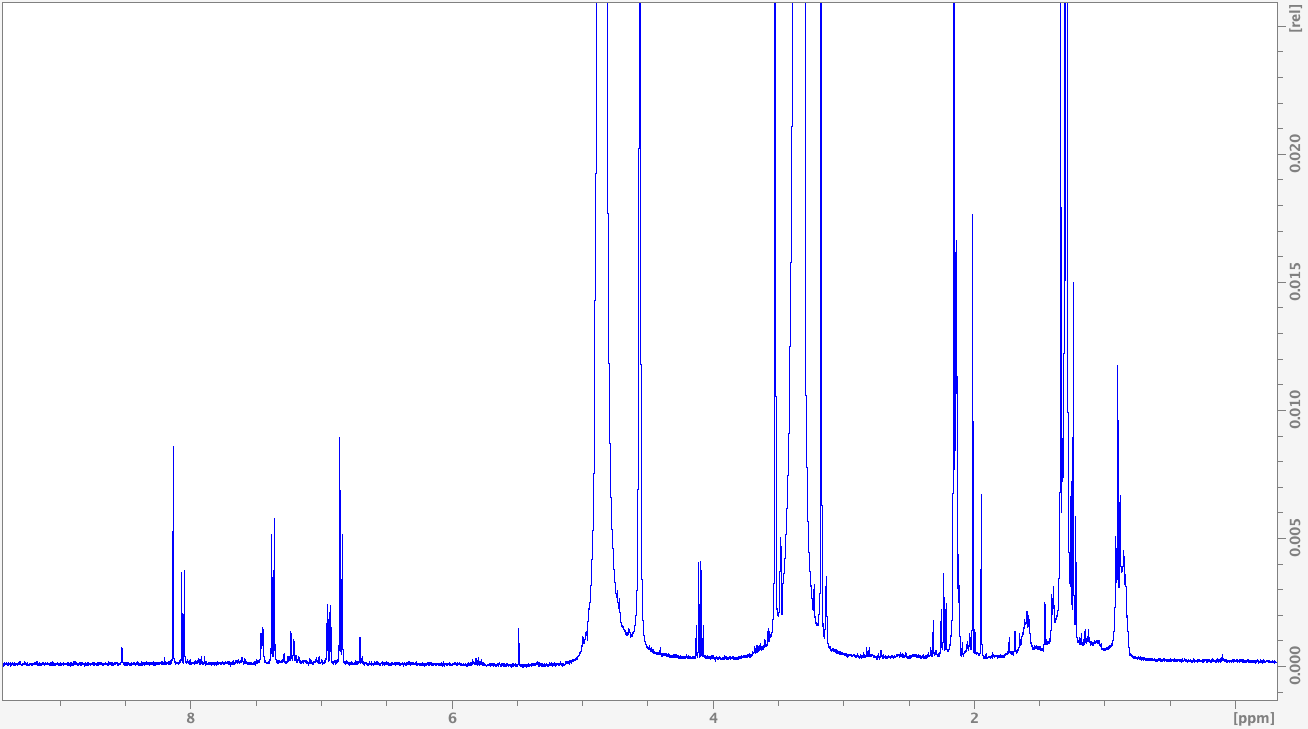
**

**Supplementary Figure 6.** ^1^H NMR of Uracil in MeOD-d4.


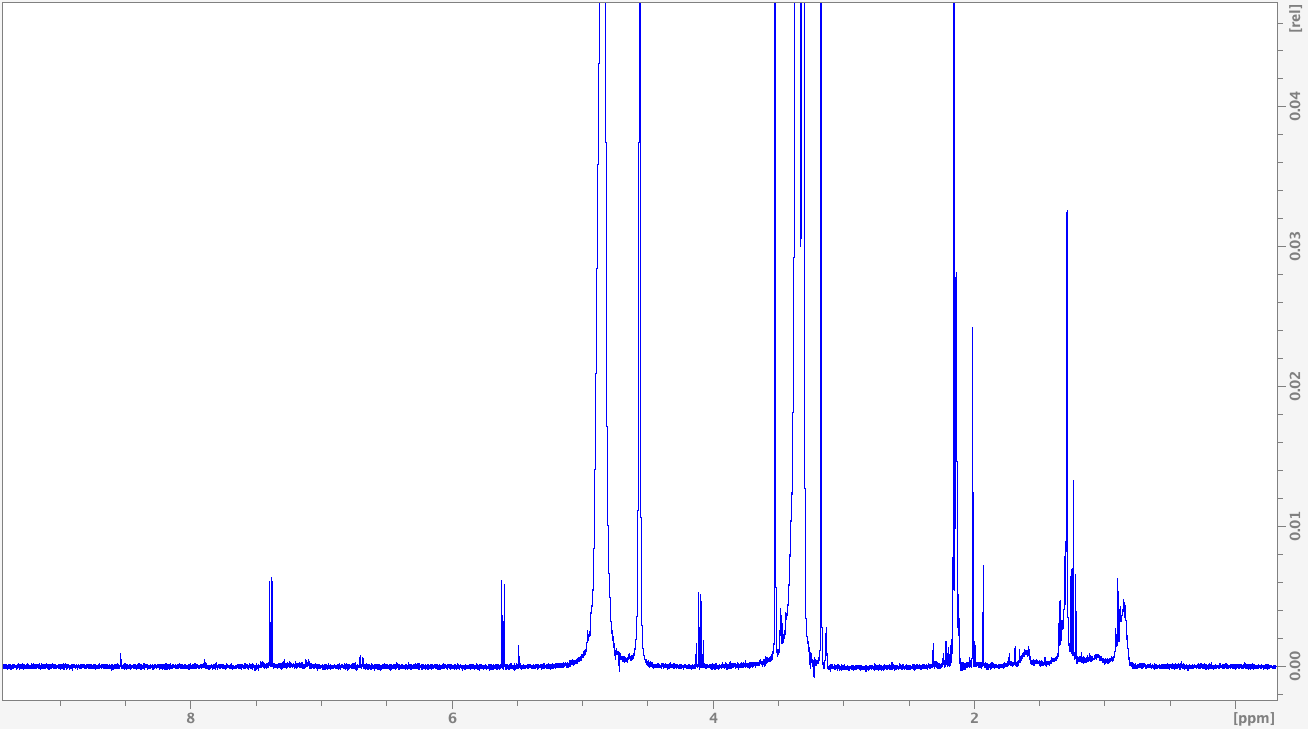


**References for Supplementary Table 2:**

Ahmad, M., El-Gendy, A., Ahmed, R., Hassan, H., El-Kabbany, H. & Merdash, A. (2017) Exploring the Antimicrobial and Antitumor Potentials of <i>Streptomyces</i> sp AGM12-1 Isolated from Egyptian Soil. *Frontiers in Microbiology*, 8.

Ahmed, R. N., Daniel, F., Gbala, I. D. & Sanni, A. (2020) Potentials of Actinomycetes from Reserved Environments as Antibacterial Agents Against Drug-Resistant Clinical Bacterial Strains. *Ethiop J Health Sci*, 30(2), 251-258.

Alghamdi, A. a. A. T. a. K. N. a. A. Y. (2021) Evaluating Desert Actinomycetes for Enzyme and Antibacterial Production. *Journal of Pure and Applied Microbiology*, 15.

Alhadrami, H. A., Sayed, A. M., Al-Khatabi, H., Alhakamy, N. A. & Rateb, M. E. (2021) Scaffold Hopping of α-Rubromycin Enables Direct Access to FDA-Approved Cromoglicic Acid as a SARS-CoV-2 M. *Pharmaceuticals (Basel)*, 14(6).

Arn, F., Frasson, D., Kroslakova, I., Rezzonico, F., Pothier, J. F., Riedl, R. & Sievers, M. (2020) Isolation and Identification of Actinomycetes Strains from Switzerland and their Biotechnological Potential. *Chimia (Aarau)*, 74(5), 382-390.

Ashraf, N., Bechthold, A., Anwar, M. A., Ghauri, M. A., Anjum, M. S., Khan, A. N., Akhtar, K. & Khaliq, S. (2021) Production of a broad spectrum streptothricin like antibiotic from halotolerant Streptomyces fimbriatus isolate G1 associated with marine sediments. *Folia Microbiol (Praha)*, 66(4), 639-649.

Aslam, S. a. S. W. a. S. I. (2020) Taxonomic Diversity, Antimicrobial Potential and Metabolite Profiling of Aquatic Actinobacteria from Kallar Kahar Lake, Pakistan. *Pakistan Journal of Zoology*, 52.

Ayswarya, S. (2020) Antimicrobial and Antitubercular Activity of Endophytic Actinobacterium and Streptomyces Sp. SACC 4 Isolated from the Mangrove Plant, Rhizophora apiculate. *Bioscience Biotechnology Research Communications*, 13, 1050-1054.

Benreguieg, M. a. A. D. E. H. a. H. K. a. M. h. K. (2017) Bioactive compound produced from Algerian arid soil Streptomyces MBA07 and its antimicrobial activity. *Bioscience Research*, 14, 678-685.

Bhosale, H., Kadam, T., Mirajgave, R. & Holkar, S. (2018) Optimization and characterization of antifungal metabolite from a soil actinomycete <i>Streptomyces indiaensis</i> SRT1. *Indian Journal of Biotechnology*, 17(2), 261-271.

Bommareddy, S. K. a. K. P. S. a. S. D. K. (2018) Screening and Identification of Novel Isolate Streptomyces sp., NLKPB45 from Nellore costal region for its Biomedical Applications. *Saudi Journal of Biological Sciences*, 26.

Bundale, S., Singh, J., Begde, D., Nashikkar, N. & Upadhyay, A. (2019) Rare actinobacteria: a potential source of bioactive polyketides and peptides. *World J Microbiol Biotechnol*, 35(6), 92.

Cao, T. a. N. T.-L. a. T. V.-H. a. M. H. a. V.-T. Q. a. N. M.-A. a. L.-T. H.-M. a. C. V.-M. a. (2019) Synthesis, Structure and Antimicrobial Activity of Novel Metabolites from a Marine Actinomycete in Vietnam's East Sea. *Natural Product Communications*, 14, 1934578X1901400.

Cheema, M. T., Ponomareva, L. V., Liu, T., Voss, S. R., Thorson, J. S., Shaaban, K. A. & Sajid, I. (2021) Taxonomic and Metabolomics Profiling of Actinobacteria Strains from Himalayan Collection Sites in Pakistan. *Curr Microbiol*, 78(8), 3044-3057.

Chemoh, W., Bin-Ismail, W. & Dueramae, S. (2021) Antagonistic Potential of Soil. *Int J Microbiol*, 2021, 2545441.

Chen, C., Ye, Y., Wang, R., Zhang, Y., Wu, C., Debnathl, S., Ma, Z., Wang, J. & Wu, M. (2018a) <i>Streptomyces nigra</i> sp.nov Is a Novel Actinobacterium Isolated From Mangrove Soil and Exerts a Potent Antitumor Activity <i>in Vitro</i>. *Frontiers in Microbiology*, 9.

Chen, L., Wang, X. N., Fu, C. M. & Wang, G. Y. (2019) Phylogenetic Analysis and Screening of Antimicrobial and Antiproliferative Activities of Culturable Bacteria Associated with the Ascidian. *Biomed Res Int*, 2019, 7851251.

Chen, L. a. W. Z. a. D. S. a. W. G.-y. (2021) Antimicrobial Activity and Functional Genes of Actinobacteria from Coastal Wetland. *Current Microbiology*, 78, 1-10.

Chen, Y., Shafi, J., Li, M., Fu, D. & Ji, M. (2018b) Insecticidal activity of endophytic actinomycetes isolated from <i>Azadirachta indica</i> against <i>Myzus persicae</i>. *Archives of Biological Sciences*, 70(2), 349-357.

Choi, H., Lee, W., Kim, E., Ku, S. K. & Bae, J. S. (2019) Inhibitory effects of collismycin C and pyrisulfoxin A on particulate matter-induced pulmonary injury. *Phytomedicine*, 62, 152939.

Dhaneesha, M., Benjamin Naman, C., Krishnan, K. P., Sinha, R. K., Jayesh, P., Joseph, V., Bright Singh, I. S., Gerwick, W. H. & Sajeevan, T. P. (2017) Streptomyces artemisiae MCCB 248 isolated from Arctic fjord sediments has unique PKS and NRPS biosynthetic genes and produces potential new anticancer natural products. *3 Biotech*, 7(1), 32.

Fang, Q., Maglangit, F., Wu, L., Ebel, R., Kyeremeh, K., Andersen, J. H., Annang, F., Pérez-Moreno, G., Reyes, F. & Deng, H. (2020) Signalling and Bioactive Metabolites from. *Molecules*, 25(3).

Fang, X., Shen, J., Wang, J., Chen, Z., Iin, P., Chen, Z., Liu, L., Zeng, H. & Jin, X. (2018) Antifungal activity of 3-acetylbenzamide produced by actinomycete WA23-4-4 from the intestinal tract of <i>Periplaneta americana</i>. *Journal of Microbiology*, 56(7), 516-523.

Fatima, A., Aftab, U., Shaaban, K. A., Thorson, J. S. & Sajid, I. (2019) Spore forming Actinobacterial diversity of Cholistan Desert Pakistan: Polyphasic taxonomy, antimicrobial potential and chemical profiling. *BMC Microbiol*, 19(1), 49.

Fatima, A. a. R. S. a. S. I. (2018) Anti-MRSA potential and metabolic fingerprinting of actinobacteria from Cholistan desert, Pakistan. *Tropical Journal of Pharmaceutical Research*, 17.

Fatin, S., Boon-Khai, T., Shu-Chien, A., Khairuddean, M. & Abdullah, A. (2017) A Marine Actinomycete Rescues <i>Caenorhabditis elegans</i> from <i>Pseudomonas aeruginosa</i> Infection through Restitution of <i>Lysozyme 7</i>. *Frontiers in Microbiology*, 8.

Ganesan, P. a. R. D. a. H. R. a. D. H. A. a. R. G. M. a. G. P. M. a. A.-D. A. a. I. S. (2016) Antimicrobial activity of some actinomycetes from Western Ghats of Tamil Nadu, India-NC-ND license (<http://creativecommons.org/licenses/by-nc-nd/4.0/>). *Alexandria Journal of Medicine*, 53.

Gomathi, A. & Gothandam, K. (2019) Investigation of anti-inflammatory and toxicity effects of mangrove-derived <i>Streptomyces rochei</i> strain VITGAP173. *Journal of Cellular Biochemistry*, 120(10), 17080-17097.

Gozari, M., Bahador, N., Jassbi, A., Mortazavi, M. & Eftekhar, E. (2018) Antioxidant and cytotoxic activities of metabolites produced by a new marine <i>Streptomyces</i> sp isolated from the sea cucumber <i>Holothuria leucospilota</i>. *Iranian Journal of Fisheries Sciences*, 17(2), 413-426.

Gurovic, M. & Olivera, N. (2017) Antibacterial producing actinomycetes from Extra Andean Patagonia. *Journal of Arid Environments*, 144, 216-219.

Hamed, A. a. A.-R. A. a. F. M. a. W. D. a. E.-H. A. a. I. T. a. K. J. a. S. N. a. S. M. (2017) N-Acetylborrelidin B: A new bioactive metabolite from Streptomyces mutabilis sp. MII. *Zeitschrift für Naturforschung C*, 73.

Handayani, I., Saad, H., Ratnakomala, S., Lisdiyanti, P., Kusharyoto, W., Krause, J., Kulik, A., Wohlleben, W., Aziz, S., Gross, H., Gavriilidou, A., Ziemert, N. & Mast, Y. (2021) Mining Indonesian Microbial Biodiversity for Novel Natural Compounds by a Combined Genome Mining and Molecular Networking Approach. *Mar Drugs*, 19(6).

Hou, L., Li, Y., Wu, Q., Li, M., Older, E. A., Tang, X., Nagarkatti, P., Nagarkatti, M., Liu, Y., Li, L., Fan, D., Bugni, T. S., Shang, Z. & Li, J. (2021) Discovery of anti-infective adipostatins through bioactivity-guided isolation and heterologous expression of a type III polyketide synthase. *Bioorg Chem*, 112, 104925.

Jacob, J., Rajendran, R., Priya, S., Purushothaman, J. & Amma, D. (2017) Enhanced antibacterial metabolite production through the application of statistical methodologies by a Streptomyces nogalater NIIST A30 isolated from Western Ghats forest soil. *Plos One*, 12(4).

Jiang, Z. B., Ren, W. C., Shi, Y. Y., Li, X. X., Lei, X., Fan, J. H., Zhang, C., Gu, R. J., Wang, L. F., Xie, Y. Y. & Hong, B. (2018a) Structure-based manual screening and automatic networking for systematically exploring sansanmycin analogues using high performance liquid chromatography tandem mass spectroscopy. *J Pharm Biomed Anal*, 158, 94-105.

Jiang, Z. K., Tuo, L., Huang, D. L., Osterman, I. A., Tyurin, A. P., Liu, S. W., Lukyanov, D. A., Sergiev, P. V., Dontsova, O. A., Korshun, V. A., Li, F. N. & Sun, C. H. (2018b) Diversity, Novelty, and Antimicrobial Activity of Endophytic Actinobacteria From Mangrove Plants in Beilun Estuary National Nature Reserve of Guangxi, China. *Front Microbiol*, 9, 868.

Jose, P. & Jha, B. (2017) Intertidal marine sediment harbours Actinobacteria with promising bioactive and biosynthetic potential. *Scientific Reports*, 7.

Kaari, M., Joseph, J., Manikkam, R., Sreenivasan, A., Venugopal, G., Alexander, B. & Krishnan, S. (2022) Anti-Biofilm Activity and Biocontrol Potential of. *Indian J Microbiol*, 62(1), 32-39.

Kamarudheen, N. & Rao, K. V. B. (2019) Fatty acyl compounds from marine Streptomyces griseoincarnatus strain HK12 against two major bio-film forming nosocomial pathogens; an in vitro and in silico approach. *Microb Pathog*, 127, 121-130.

Katif, C. a. C. T. a. S. B. a. B. Z. a. H. A. a. H. T. a. N. J. a. M. B. (2022) Isolation and Structure Elucidation of Desferrioxamine B and the New Desferrioxamine B2 Antibiotics from a Brown Marine Macroalga Carpodesmia tamariscifolia Associated Streptomyces Isolate. *Biointerface Research in Applied Chemistry*, 12, 5647-5662.

Kemung, H. M., Tan, L. T., Chan, K. G., Ser, H. L., Law, J. W., Lee, L. H. & Goh, B. H. (2020) Antioxidant Activities of. *Biomed Res Int*, 2020, 6402607.

Khadayat, K., Sherpa, D. D., Malla, K. P., Shrestha, S., Rana, N., Marasini, B. P., Khanal, S., Rayamajhee, B., Bhattarai, B. R. & Parajuli, N. (2020) Molecular Identification and Antimicrobial Potential of. *Int J Microbiol*, 2020, 8817467.

Kim, J. H., Choi, J. Y., Park, D. H., Park, D. J., Park, M. G., Kim, S. Y., Ju, Y. J., Kim, J. Y., Wang, M., Kim, C. J. & Je, Y. H. (2020) Isolation and characterization of the insect growth regulatory substances from actinomycetes. *Comp Biochem Physiol C Toxicol Pharmacol*, 228, 108651.

Krishnan, K. a. M. A. (2018) Structural Elucidation and Identification of 2-Hydroxy Benzoic Acid: An Antibacterial and Cytotoxic Compound from Streptomyces sp VITHM1 Isolated from Marine Sediment Sample of Alappuzha Beach, Kerala, India. *Arabian Journal for Science and Engineering*, 43(7), 3339-3348.

Kuncharoen, N. a. F. W. a. M. M. a. S. K. a. T. S. (2019) Diversity and Antimicrobial Activity of Endophytic Actinomycetes Isolated from Plant Roots in Thailand. *Microbiology*, 88, 479-488.

Kurnianto, M. A., Kusumaningrum, H. D. & Lioe, H. N. (2020) Characterization of. *Int J Microbiol*, 2020, 8851947.

Law, J. W., Chan, K. G., He, Y. W., Khan, T. M., Ab Mutalib, N. S., Goh, B. H. & Lee, L. H. (2019) Diversity of Streptomyces spp. from mangrove forest of Sarawak (Malaysia) and screening of their antioxidant and cytotoxic activities. *Sci Rep*, 9(1), 15262.

le Roes-Hill, M., Prins, A. & Meyers, P. R. (2018) Streptomyces swartbergensis sp. nov., a novel tyrosinase and antibiotic producing actinobacterium. *Antonie Van Leeuwenhoek*, 111(4), 589-600.

Leimer, N., Wu, X., Imai, Y., Morrissette, M., Pitt, N., Favre-Godal, Q., Iinishi, A., Jain, S., Caboni, M., Leus, I. V., Bonifay, V., Niles, S., Bargabos, R., Ghiglieri, M., Corsetti, R., Krumpoch, M., Fox, G., Son, S., Klepacki, D., Polikanov, Y. S., Freliech, C. A., McCarthy, J. E., Edmondson, D. G., Norris, S. J., D'Onofrio, A., Hu, L. T., Zgurskaya, H. I. & Lewis, K. (2021) A selective antibiotic for Lyme disease. *Cell*, 184(21), 5405-5418.e16.

Li, K., Liang, Z., Chen, W., Luo, X., Fang, W., Liao, S., Lin, X., Yang, B., Wang, J., Tang, L., Liu, Y. & Zhou, X. (2019) Iakyricidins A-D, Antiproliferative Piericidin Analogues Bearing a Carbonyl Group or Cyclic Skeleton from. *J Org Chem*, 84(19), 12626-12631.

Lim, S. S. a. S. A. a. N. Z. Y. a. M. S. a. C. P. C. H. a. L. R. (2018) Isolation of actinomycetes with antibacterial activity against multi-drug resistant bacteria. *Malaysian Journal of Microbiology*, 14, 293-305.

Liu, M. J., Hwang, B. S., Jin, C. Z., Li, W. J., Park, D. J., Seo, S. T. & Kim, C. J. (2019) Screening, isolation and evaluation of a nematicidal compound from actinomycetes against the pine wood nematode, Bursaphelenchus xylophilus. *Pest Manag Sci*, 75(6), 1585-1593.

Lotfy, M. M., Hassan, H. M., Mohammed, R., Hetta, M., El-Gendy, A. O., Rateb, M. E., Zaki, M. A. & Gamaleldin, N. M. (2019) Chemical Profiling and Biological Screening of Some River Nile Derived-Microorganisms. *Front Microbiol*, 10, 787.

Mahfouz, A. (2021) Isolation, characterization, and screening of actinomycetes producing bioactive compounds from Egyptian soil. *Egyptian Pharmaceutical Journal*, 19, 381-390.

Malmierca, M. G., González-Montes, L., Pérez-Victoria, I., Sialer, C., Braña, A. F., García Salcedo, R., Martín, J., Reyes, F., Méndez, C., Olano, C. & Salas, J. A. (2018) Searching for Glycosylated Natural Products in Actinomycetes and Identification of Novel Macrolactams and Angucyclines. *Front Microbiol*, 9, 39.

Mangzira Kemung, H., Tan, L. T., Chan, K. G., Ser, H. L., Law, J. W., Lee, L. H. & Goh, B. H. (2020) sp. Strain MUSC 125 from Mangrove Soil in Malaysia with Anti-MRSA, Anti-Biofilm and Antioxidant Activities. *Molecules*, 25(15).

Manimaran, M., Rajkumar, T., Vimal, S., Taju, G., Majeed, S., Hameed, A. & Kannabiran, K. (2018) Antiviral activity of 9(10H)-Acridanone extracted from marine <i>Streptomyces fradiae</i> strain VITMK2 in <i>Litopenaeus vannamei</i> infected with white spot syndrome virus. *Aquaculture*, 488, 66-73.

Masand, M., Sivakala, K. K., Menghani, E., Thinesh, T., Anandham, R., Sharma, G., Sivakumar, N., Jebakumar, S. R. D. & Jose, P. A. (2018) Biosynthetic Potential of Bioactive Streptomycetes Isolated From Arid Region of the Thar Desert, Rajasthan (India). *Front Microbiol*, 9, 687.

Mehetre, G. T., J S, V., Burkul, B. B., Desai, D., B, S., Dharne, M. S. & Dastager, S. G. (2019) Bioactivities and molecular networking-based elucidation of metabolites of potent actinobacterial strains isolated from the Unkeshwar geothermal springs in India. *RSC Adv*, 9(17), 9850-9859.

Meng-Xi, L. I., Hui-Bin, H., Jie-Yun, L., Jing-Xiao, C. A. O. & Zhen-Wang, Z. (2021) Antibacterial Performance of a Streptomyces spectabilis Strain Producing Metacycloprodigiosin. *Curr Microbiol*, 78(7), 2569-2576.

Mohamed, H., Miloud, B., Zohra, F., García-Arenzana, J. M., Veloso, A. & Rodríguez-Couto, S. (2017) Isolation and Characterization of Actinobacteria from Algerian Sahara Soils with Antimicrobial Activities. *Int J Mol Cell Med*, 6(2), 109-120.

Nafis, A., Elhidar, N., Oubaha, B., Samri, S. E., Niedermeyer, T., Ouhdouch, Y., Hassani, L. & Barakate, M. (2018) Screening for Non-polyenic Antifungal Produced by Actinobacteria from Moroccan Habitats: Assessment of Antimycin A19 Production by. *Int J Mol Cell Med*, 7(2), 133-145.

Nandhini, S. a. S. S. a. V. A. a. S. M. (2018) Isolation, Identification and Extraction of antimicrobial compounds produced by Streptomyces sps from terrestrial soil. *Biocatalysis and Agricultural Biotechnology*, 15.

Nithya, K., Muthukumar, C., Biswas, B., Alharbi, N. S., Kadaikunnan, S., Khaled, J. M. & Dhanasekaran, D. (2018) Desert actinobacteria as a source of bioactive compounds production with a special emphases on Pyridine-2,5-diacetamide a new pyridine alkaloid produced by Streptomyces sp. DA3-7. *Microbiol Res*, 207, 116-133.

Norouzi, H., Danesh, A., Mohseni, M. & Rabbani Khorasgani, M. (2018) Marine Actinomycetes with Probiotic Potential and Bioactivity against Multidrug-resistant Bacteria. *Int J Mol Cell Med*, 7(1), 44-52.

Odumosu, B., Buraimoh, O., Okeke, C., Ogah, J. & Michel, F. (2017) Antimicrobial activities of the <i>Streptomyces ceolicolor</i> strain AOB KF977550 isolated from a tropical estuary. *Journal of Taibah University For Science*, 11(6), 836-841.

Pachaiyappan, S. K. a. G. P. M. a. I. S. a. A.-D. N. a. S. D. (2017) In vitro antagonistic activity of soil Streptomyces Collinus Dpr20 against bacterial pathogens. *Journal of Microbiology, Biotechnology and Food Sciences*, 7, 317-324.

Pachaiyappan, S. K. a. L. C. a. Z. Z. a. D. Y. a. S. C. a. S. Y. a. W. N.-K. a. J. J. (2020) Chemical Diversity of Metabolites and Antibacterial Potential of Actinomycetes Associated with Marine Invertebrates from Intertidal Regions of Daya Bay and Nansha Islands. *Microbiology*, 89, 483-492.

Paderog, M. J. V., Suarez, A. F. L., Sabido, E. M., Low, Z. J., Saludes, J. P. & Dalisay, D. S. (2020) Anthracycline Shunt Metabolites From Philippine Marine Sediment-Derived. *Front Microbiol*, 11, 743.

Pavan Kumar, J. G. S., Gomathi, A., Gothandam, K. M. & Vasconcelos, V. (2018) Bioactivity Assessment of Indian Origin-Mangrove Actinobacteria against Candida albicans. *Mar Drugs*, 16(2).

Peng, F., Zhang, M. Y., Hou, S. Y., Chen, J., Wu, Y. Y. & Zhang, Y. X. (2020) Insights into Streptomyces spp. isolated from the rhizospheric soil of Panax notoginseng: isolation, antimicrobial activity and biosynthetic potential for polyketides and non-ribosomal peptides. *BMC Microbiol*, 20(1), 143.

Phongsopitanun, W. a. S. P. a. R. K. a. P. R. a. P. P. a. T. S. (2020) Diversity and antimicrobial activity of culturable endophytic actinobacteria associated with Acanthaceae plants. *ScienceAsia*, 46, 288.

Qi, D., Zou, L., Zhou, D., Chen, Y., Gao, Z., Feng, R., Zhang, M., Li, K., Xie, J. & Wang, W. (2019) Taxonomy and Broad-Spectrum Antifungal Activity of. *Front Microbiol*, 10, 1390.

Quintero, M., Velásquez, A., Jutinico, L. M., Jiménez-Vergara, E., Blandón, L. M., Martinez, K., Lee, H. S. & Gómez-León, J. (2018) Bioprospecting from marine coastal sediments of Colombian Caribbean: screening and study of antimicrobial activity. *J Appl Microbiol*, 125(3), 753-765.

Ravi, L. & Kannabiran, K. (2018) Extraction and Identification of Gancidin W from Marine <i>Streptomyces</i> sp. VITLGK012. *Indian Journal of Pharmaceutical Sciences*, 80(6), 1093-1099.

Ravi, L. a. R. A. a. K. K. (2017) Marine Streptomyces paradoxus VITALK03 derived gancidin W mediated cytotoxicity through Ras-Raf-MEK-ERK signalling pathway. *Indian Journal of Biotechnology*, 16, 164-175.

Reegan, A. D., Kumar, P. S., Asharaja, A. C., Devi, C., Jameela, S., Balakrishna, K. & Ignacimuthu, S. (2021) Larvicidal and ovicidal activities of phenyl acetic acid isolated from Streptomyces collinus against Culex quinquefasciatus Say and Aedes aegypti L. (Diptera: Culicidae). *Exp Parasitol*, 226-227, 108120.

Ribeiro, I., Girão, M., Alexandrino, D. A. M., Ribeiro, T., Santos, C., Pereira, F., Mucha, A. P., Urbatzka, R., Leão, P. N. & Carvalho, M. F. (2020) Diversity and Bioactive Potential of Actinobacteria Isolated from a Coastal Marine Sediment in Northern Portugal. *Microorganisms*, 8(11).

Sajjad, W., Ahmad, S., Aziz, I., Azam, S. S., Hasan, F. & Shah, A. A. (2018) Antiproliferative, antioxidant and binding mechanism analysis of prodigiosin from newly isolated radio-resistant Streptomyces sp. strain WMA-LM31. *Mol Biol Rep*, 45(6), 1787-1798.

Sapkota, A., Thapa, A., Budhathoki, A., Sainju, M., Shrestha, P. & Aryal, S. (2020) Isolation, Characterization, and Screening of Antimicrobial-Producing Actinomycetes from Soil Samples. *Int J Microbiol*, 2020, 2716584.

Sebak, M., Saafan, A. E., AbdelGhani, S., Bakeer, W., El-Gendy, A. O., Espriu, L. C., Duncan, K. & Edrada-Ebel, R. (2019) Bioassay- and metabolomics-guided screening of bioactive soil actinomycetes from the ancient city of Ihnasia, Egypt. *PLoS One*, 14(12), e0226959.

Shah, A., Shakeel-u-Rehman, Hussain, A., Mushtaq, S., Rather, M., Shah, A., Ahmad, Z., Khan, I., Bhat, K. & Hassan, Q. (2017) Antimicrobial investigation of selected soil actinomycetes isolated from unexplored regions of Kashmir Himalayas, India. *Microbial Pathogenesis*, 110, 93-99.

Sharma, A., Kaushik, N., Bajaj, A., Rasane, M., Shouche, Y. S., Marzouk, T. & Djébali, N. (2021) Screening of Tomato Seed Bacterial Endophytes for Antifungal Activity Reveals Lipopeptide Producing. *Front Microbiol*, 12, 609482.

Sharma, M. & Manhas, R. K. (2019) Purification and characterization of actinomycins from Streptomyces strain M7 active against methicillin resistant Staphylococcus aureus and vancomycin resistant Enterococcus. *BMC Microbiol*, 19(1), 44.

Sharma, N. a. M. R. a. B. R. a. O. P. (2021) Bioefficacy of Bio-metabolites Produced by Streptomyces sp. Strain MR-14 in Ameliorating Meloidogyne incognita Stress in Solanum lycopersicum Seedlings. *Journal of Plant Growth Regulation*, 41, 1-13.

Sharma, R. a. M. P. a. J. T. a. B. S. a. S. B. (2022) Screening of Secondary Metabolites Produced by Streptomyces Species from a Soil Sample that Can Produce Anti-Nematodal and Antiprotozoal Avermectins. *Journal of Pharmaceutical Research International*, 33, 90-98.

Shrestha, B., Nath, D. K., Maharjan, A., Poudel, A., Pradhan, R. N. & Aryal, S. (2021) Isolation and Characterization of Potential Antibiotic-Producing Actinomycetes from Water and Soil Sediments of Different Regions of Nepal. *Int J Microbiol*, 2021, 5586165.

Siddharth, S., Vittal, R. R., Wink, J. & Steinert, M. (2020) Diversity and Bioactive Potential of Actinobacteria from Unexplored Regions of Western Ghats, India. *Microorganisms*, 8(2).

Singh, V., Haque, S., Khare, S., Tiwari, A., Katiyar, D., Banerjee, B., Kumari, K. & Tripathi, C. (2018) Isolation and purification of antibacterial compound from <i>Streptomyces levis</i> collected from soil sample of north India. *Plos One*, 13(7).

Somasundaram, S. a. N. S. a. A. V. a. B. N. (2020) Screening of Bioactive Compounds from Marine Actinomycetes, 01.

Sproule, A., Correa, H., Decken, A., Haltli, B., Berrué, F., Overy, D. P. & Kerr, R. G. (2019) Terrosamycins A and B, Bioactive Polyether Ionophores from. *Mar Drugs*, 17(6).

Sundaramanickam, A. a. V. T. a. S. S. a. B. T. (2019) Screening and fractional purification of antimicrobial compound of Streptomyces sp. MAB 18 isolated from coastal sediment of Nagapattinam, south-east coast of India. *Indian Journal of Geo-Marine Sciences*, 48(5), 662-669.

Uttara, V. (2020) Bioactive metabolites produced from Streptomyces enissocaesilis SSASC10 against fish pathogens. *Biocatalysis and Agricultural Biotechnology*, 29, 101802.

Wang, H., Sun, T., Song, W., Guo, X., Cao, P., Xu, X., Shen, Y. & Zhao, J. (2020) Taxonomic Characterization and Secondary Metabolite Analysis of NEAU-wh3-1: An. *Microorganisms*, 8(3).

Wang, R. J., Zhang, S. Y., Ye, Y. H., Yu, Z., Qi, H., Zhang, H., Xue, Z. L., Wang, J. D. & Wu, M. (2019) Three New Isoflavonoid Glycosides from the Mangrove-Derived Actinomycete. *Mar Drugs*, 17(5).

Wibowo, J. T., Kellermann, M. Y., Versluis, D., Putra, M. Y., Murniasih, T., Mohr, K. I., Wink, J., Engelmann, M., Praditya, D. F., Steinmann, E. & Schupp, P. J. (2019) Biotechnological Potential of Bacteria Isolated from the Sea Cucumber. *Mar Drugs*, 17(11).

Wu, G., Nielson, J. R., Peterson, R. T. & Winter, J. M. (2017) Bonnevillamides, Linear Heptapeptides Isolated from a Great Salt Lake-Derived Streptomyces sp. *Mar Drugs*, 15(7).

Wu, Q., Zhang, G., Wang, B., Li, X., Yue, S., Chen, J., Zhang, H. & Wang, H. (2018) Production and Identification of Inthomycin B Produced by a Deep-Sea Sediment-Derived <i>Streptomyces</i> sp YB104 Based on Cultivation-Dependent Approach. *Current Microbiology*, 75(7), 942-951.

Yang, Y., Zhang, S. W. & Li, K. T. (2019) Antagonistic activity and mechanism of an isolated Streptomyces corchorusii stain AUH-1 against phytopathogenic fungi. *World J Microbiol Biotechnol*, 35(9), 145.

Zhang, S., Zhang, L., Zhu, J., Chen, H., Chen, Z., Si, T. & Liu, T. (2021) Genomic and Metabolomic Investigation of a Rhizosphere Isolate. *Molecules*, 26(8).

Zhao, H., Chen, X., Zhu, Y., Kong, Y., Zhang, S., Deng, X., Ouyang, P., Zhang, W., Hou, S., Wang, X. & Xie, T. (2020) New peptidendrocins and anticancer chartreusin from an endophytic bacterium of. *Ann Transl Med*, 8(7), 455.

Zhou, B., Hu, Z. J., Zhang, H. J., Li, J. Q., Ding, W. J. & Ma, Z. J. (2019) Bioactive staurosporine derivatives from the Streptomyces sp. NB-A13. *Bioorg Chem*, 82, 33-40.
